# HHEX-PRKAR2B axis-mediated PKA activation drives glucose metabolism-dependent progression of pancreatic ductal adenocarcinoma

**DOI:** 10.1016/j.isci.2026.114691

**Published:** 2026-01-14

**Authors:** Junxiang Wen, Qiuchen Li, Shuxiang Xu, Wenjun Lu, Fei Wu, Jiatao Lou, Lin Wang

**Affiliations:** 1 Department of Laboratory Medicine, Shanghai General Hospital, Shanghai Jiao Tong University School of Medicine, Shanghai 200080, China; 2 College of Health Science and Technology, Shanghai Jiao Tong University School of Medicine, Shanghai, China

**Keywords:** Human metabolism, Molecular interaction, Cancer

## Abstract

By virtue of its function as a key metabolic regulator, malignant transformation in the pancreas not only confers high aggressiveness but also disrupts systemic metabolism. However, the causal relationship between metabolic reprogramming and the progression of pancreatic ductal adenocarcinoma (PDAC) remains incompletely understood. This study identifies aberrant protein kinase A (PKA) activation in PDAC, correlating with poor prognosis. Mechanistically, downregulation of the transcription factor hematopoietically expressed homeobox (HHEX) represses protein kinase cAMP-dependent type II regulatory subunit beta (PRKAR2B), relieving inhibition on PKA catalytic activity. A high-glucose microenvironment promotes cAMP production, further activating PKA, which then enhances glycolysis via specific upregulation of hexokinase 2 (HK2). *In vivo*, high glucose synergized with PKA activation to promote metastasis, whereas glycolysis inhibition blocked new metastases. Thus, HHEX-PRKAR2B-mediated PKA activation is a critical hub for PDAC progression, modulated by glucose and reinforcing glycolysis via HK2, revealing novel therapeutic targets for metabolic intervention.

## Highlights

• The HHEX-PRKAR2B-PKA-glycolysis metabolic-signaling axis drives PDAC progression
• PRKAR2B suppression and cAMP elevation sustain PKA activation in PDAC
• PKA activity is a potential biomarker for PDAC differentiation grade and poor prognosis
• PRKAR2B enables targeted suppression of PDAC progression as a promising therapeutic target

## Graphical abstract

**Figure:**
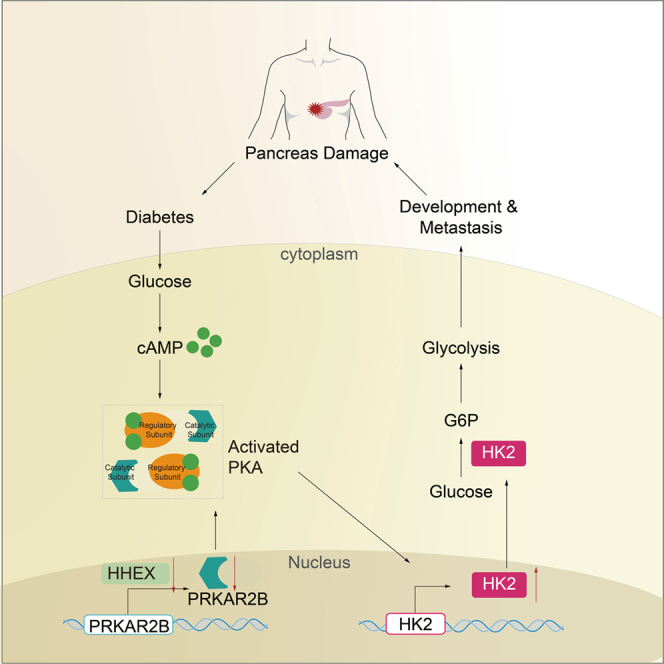

## Introduction

Pancreatic ductal adenocarcinoma (PDAC) is one of the most aggressive malignancies of the digestive tract, with a 5-year survival rate below 10%.^1^ This poor prognosis is largely attributable to its tendency for early metastasis and inherent resistance to therapy.^2^ Notably, as the pancreas plays a central role in systemic metabolic regulation, its malignant transformation profoundly disrupts whole-body metabolic homeostasis.^3^ Studies have shown that the prevalence of diabetes mellitus in pancreatic cancer patients is 47%, of which 27% are new-onset diabetes. Notably, up to 80% of these new-onset diabetes cases are diagnosed while the pancreatic cancer is still asymptomatic,^4^ strongly suggesting a close association between disordered glucose metabolism and the development of pancreatic cancer. Basic research has revealed that a high-glucose microenvironment promotes tumor progression and immune evasion by inducing *Kirsten rat sarcoma (KRAS)* mutations through insufficient deoxynucleoside triphosphate supply, and facilitating lactate-dependent modification of methyltransferase 16.^5^ Therefore, elucidating the molecular mechanisms by which glucose metabolism reprogramming drives malignant progression of PDAC is essential for developing effective therapeutic strategies.

Protein kinase A (PKA), a key regulator of energy metabolism, is essential for maintaining glucose metabolic homeostasis.^6^ Yang et al. showed that PKA recruits the 14-3-3ζ-RNA polymerase II complex through serine 28 phosphorylation at histone H3, thereby activating transcription of the gluconeogenic genes *phosphoenolpyruvate carboxykinase 1* and *glucose-6-phosphatase catalytic subunit*.^7^ Furthermore, PKA promotes glycogenolysis via activation of phosphorylase kinase and simultaneously suppresses glycogen synthase activity to prevent glycogen synthesis, ensuring precise control of energy metabolism.^8^ Although the role of PKA in normal glucose metabolism is well established, its specific role in the mechanisms of reprogramming glucose metabolism and promoting malignancy in PDAC, a cancer characterized by profound metabolic dysregulation, remains elusive.

As a conserved effector of the cyclic adenosine monophosphate (cAMP) signaling pathway, PKA exhibits considerable tissue specificity; dysregulation of PKA activity has been shown to drive malignant progression in various cancers.^9^^,^^10^^,^^11^ In papillary thyroid carcinoma, for instance, *protein kinase cAMP-activated catalytic subunit alpha (PRKACA)* gene fusions encode a constitutively active form of the PKA catalytic subunit, which phosphorylates large tumor suppressor kinase 1/2 (LATS1/2) kinases to inhibit Yes1-associated transcriptional regulator nuclear translocation, thereby ameliorating metastatic potential.^12^ In contrast, in breast cancer, overexpression of the protein kinase cAMP-activated catalytic subunit beta (PRKACB) subunit promotes distant metastasis by inducing epithelial-mesenchymal transition (EMT) through cAMP responsive element binding protein 1 (CREB) phosphorylation (pCREB).^13^ Clinical cohort analyses indicate that patients with breast cancer with high PKA activity have a 41% lower metastasis-free survival rate.^14^ Nevertheless, whether PKA activity is altered in PDAC and its potential role in disease progression remain unelucidated.

This study systematically investigated the activation mechanism of the PKA pathway in PDAC and its functional crosstalk with glucose metabolism. We identified aberrant hyperactivation of the PKA signaling pathway in PDAC, which was significantly correlated with poor patient prognosis. Mechanistically, PKA activation occurs through two complementary mechanisms: downregulation of the transcription factor hematopoietically expressed homeobox (HHEX) suppresses protein kinase cAMP-dependent type II regulatory subunit beta (PRKAR2B) expression, thereby relieving inhibition on the catalytic subunit of PKA, while a high-glucose microenvironment facilitates cAMP production via enhanced ATP synthesis, further promoting PKA activation. Subsequently, PKA enhances glycolytic flux through specific upregulation of *hexokinase 2 (HK2)* transcription. These findings provide mechanistic insight into the molecular basis of PDAC progression and offer a theoretical foundation for a therapeutic strategy targeting metabolic pathways.

## Results

### Aberrant activation of PKA signaling pathway in PDAC

**Figure 1:**
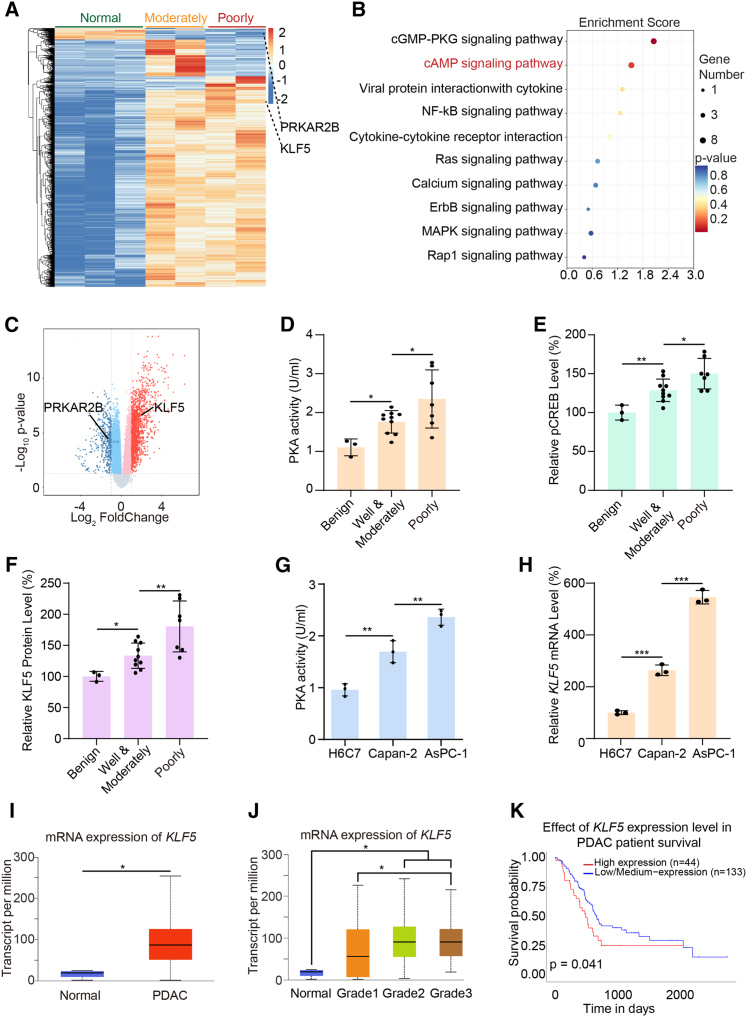
PKA signaling pathway aberrantly activated in PDAC (A) Heatmap showing mRNA expression levels of moderately and poorly differentiated PDAC from the GEO database (GSE226307). PRKAR2B and KLF5 were labeled. (B) KEGG pathway enrichment analysis of differentially expressed genes from RNA-seq in GEO database (GSE226307). The “cAMP signaling pathway” is highlighted in red. (C) Volcano plot of RNA-seq data comparing PDAC tissue and normal pancreatic tissue (GSE16515). Red dots represent upregulated mRNAs, blue dots represent downregulated mRNAs (*p* < 0.05, log_2_foldchange >1 or < –1). PRKAR2B and KLF5 were labeled. (D–F) Colorimetric analysis of PKA activity (D) and ELISA analysis of pCREB and KLF5 levels (E–F) in pancreatic puncture fluid from patients with benign pancreatic diseases (*n* = 3 independent samples), well or moderately differentiated PDAC (*n* = 10 independent samples), or poorly differentiated PDAC (*n* = 7 independent samples). Data were expressed as mean ± SD. ∗, *p* < 0.05; ∗∗, *p* < 0.01. (G and H) Colorimetric analysis of PKA activity (G) and qPCR analysis of mRNA expression of *KLF5* (H) in H6C7, Capan-2, and AsPC-1 cells (*n* = 3 independent repeats). Data were expressed as mean ± SD. ∗∗, *p* < 0.01, ∗∗∗, *p* < 0.001. (I and J) mRNA expression profiles of *KLF5* based on data from 182 PDAC patients and 4 normal controls in the TCGA database. ∗, *p* < 0.05. (K) Kaplan-Meier survival analysis of *KLF5* expression using data from 182 PDAC patients in the TCGA database. ∗, *p* < 0.05.

PDAC is one of the most lethal malignancies, largely owing to its high metastatic potential and propensity for early dissemination.^15^ Clinicopathological studies have consistently shown that patients with poorly differentiated PDAC face a significantly higher risk of invasion and metastasis compared to those with moderately or well-differentiated tumors.^16^ To uncover the molecular mechanisms underlying PDAC progression, particularly its poorly differentiated form, we analyzed RNA-sequencing data from PDAC samples of different differentiation grades available in the Gene Expression Omnibus (GEO) database (accession GSE226307). Transcriptomic analysis revealed significant expression differences among normal pancreatic tissues, poorly differentiated PDAC, and moderately or well-differentiated PDAC (Figure 1A). Subsequent Kyoto Encyclopedia of Genes and Genomes (KEGG) pathway enrichment analysis of the differentially expressed genes identified in these PDAC samples with varying differentiation degrees revealed significant enrichment of the cAMP signaling pathway (Figure 1B). To further verify the role of the cAMP signaling pathway in pancreatic cancer, we analyzed gene expression data from the GSE16515 dataset, which includes 36 primary pancreatic tumor tissues and 16 matched adjacent non-tumor tissues. A total of 1,715 differentially expressed genes were identified (Figure 1C). KEGG pathway enrichment analysis of these genes also demonstrated significant enrichment of the cAMP signaling pathway (Figure S1A). Collectively, these findings suggest that the cAMP signaling pathway may play a critical role in the pathogenesis and progression of pancreatic cancer.

cAMP, a key second messenger, regulates diverse cellular processes including proliferation, differentiation, and migration through the activation of PKA and other effectors.^17^ Although aberrant cAMP-PKA signaling has been implicated in the progression of several cancers,^18^ its activity and functional significance in the malignancy of PDAC are yet to be fully elucidated. To assess the activation status of the PKA pathway in PDAC, we first analyzed clinical samples obtained through fine-needle aspiration from 20 patients at Shanghai General Hospital, including three with benign pancreatic disease, ten with moderately or well-differentiated PDAC, and seven with poorly differentiated PDAC. A colorimetric PKA activity assay showed that PKA activity was the lowest in the benign group (Figure 1D). In contrast, poorly differentiated PDAC samples exhibited significantly higher PKA activity compared with the moderately/well-differentiated PDAC group and benign controls, respectively, with statistically significant differences among all groups (Figure 1D). Since PKA activation is known to induce phosphorylation of CREB at Ser133 and subsequently upregulate downstream targets such as Krüppel-like transcription factor 5 (KLF5),^18^^,^^19^ we further evaluated PKA pathway activity by measuring pCREB and KLF5 protein levels in the same samples using the enzyme-linked immunosorbent assay. Consistent with the PKA activity results, both pCREB and KLF5 levels were significantly elevated in the poorly differentiated PDAC group compared to the moderately/well-differentiated and benign groups (Figures 1E and 1F). In addition, analysis of PDAC and control samples obtained from the GEO database (GSE226307 and GSE16515) demonstrated significant elevation in KLF5 transcript levels (Figures 1A and 1C). Collectively, these results indicate aberrant activation of the PKA signaling pathway in PDAC, with the highest level of activation observed in poorly differentiated tumors.

The HPDE6-C7 (H6C7) cell line, which models normal human pancreatic ductal epithelium, displays a typical nuclear-to-cytoplasmic ratio, lacks expression of metastasis-associated markers such as cyclooxygenase (COX)-2 and matrix metalloproteinases, and shows no signs of malignant proliferation, consistent with the quiescent phenotype of normal ductal cells.^20^ The Capan-2 cell line, derived from a primary PDAC tumor, retains acinar structures and forms gland-like lumina, exhibits moderately high cell adhesion molecule 5 (CEA) expression with mild cellular atypia, and has intermediate invasive ability, reflecting features of moderately differentiated PDAC.^21^ In contrast, the AsPC-1 cell line, derived from metastatic ascites in a patient with PDAC, shows loss of E-cadherin, high COX-2 expression, no epithelial polarity, and significant nuclear atypia.^22^ This line efficiently generates lung and liver metastases *in vivo*, closely mimicking the behavior of advanced, poorly differentiated PDAC. Based on these traits, we used H6C7, Capan-2, and AsPC-1 as *in vitro* models representing normal pancreatic ductal epithelium, moderately differentiated PDAC, and poorly differentiated PDAC, respectively. PKA activity, measured via colorimetric assay, was significantly higher in AsPC-1 cells than in Capan-2 cells, and both PDAC lines showed elevated activity compared to H6C7 (Figure 1G). Concurrently, qPCR analysis revealed higher transcriptional levels of KLF5, a downstream target of PKA, in the same order across cell lines (Figure 1H). These *in vitro* results further support aberrant activation of the PKA signaling pathway in pancreatic cancer cells.

Finally, we analyzed PDAC samples from The Cancer Genome Atlas (TCGA) and observed significantly higher transcriptional levels of the PKA downstream target gene KLF5 in tumor tissues compared with normal adjacent tissues (Figure 1I). Moreover, KLF5 expression increased significantly with the decline in the tumor differentiation grade (Figure 1J). Consistent with these findings, analysis of the Clinical Proteomic Tumor Analysis Consortium (CPTAC) proteomics database showed marked elevation in KLF5 protein levels in PDAC samples (Figure S1B). Survival analysis revealed that high expression of KLF5 was associated with significantly shorter overall survival in patients with PDAC, suggesting that PKA activation correlates with poor prognosis (Figure 1K). Together, these results demonstrate significant activation of PKA signaling in PDAC, which is strongly associated with aggressive tumor features and adverse clinical outcomes.

### Transcriptional suppression of PRKAR2B leads to PKA activation in PDAC

**Figure 2:**
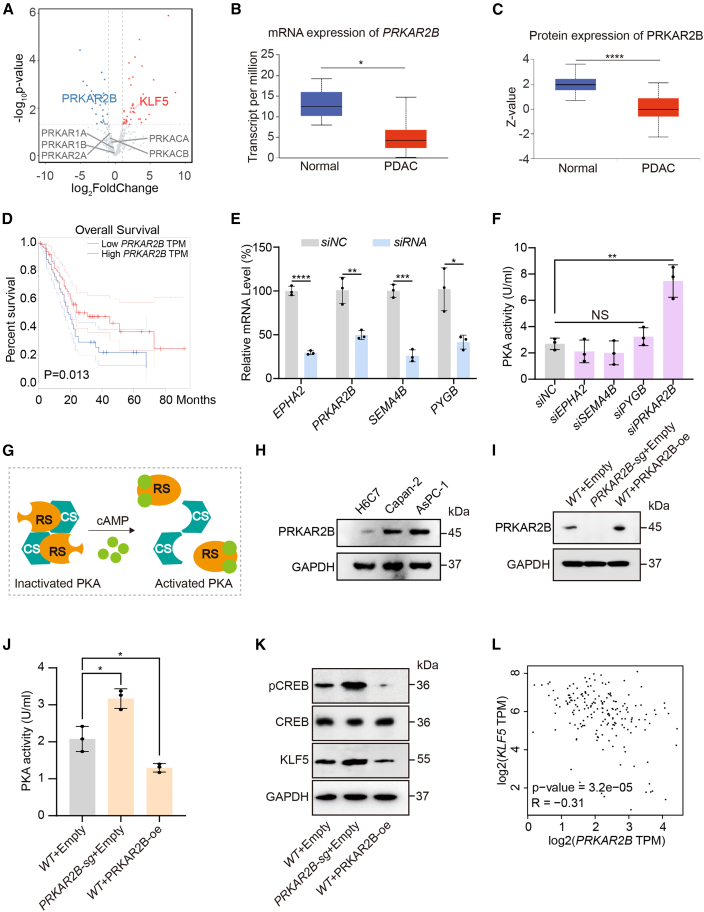
*PRKAR2B* downregulation activates PKA in PDAC (A) Volcano plot of RNA-seq data comparing PDAC tissue and normal pancreatic tissue (GSE226307). Red dots represent upregulated mRNAs, blue dots represent downregulated mRNAs (*p* < 0.05, log_2_foldchange >1 or < -1). KLF5, PRKAR1A, PRKAR1B, PRKAR2A, PRKAR2B, PRKACA, and PRKACB were labeled. (B) mRNA expression levels of *PRKAR2B* based on data from 182 PDAC patients and 4 normal controls in the TCGA database. ∗, *p* < 0.05. (C) Protein expression levels of PRKAR2B based on data from 137 PDAC patients and 74 normal controls in the CPTAC database. ∗∗∗∗, *p* < 0.0001. (D) Kaplan-Meier survival analysis of *PRKAR2B* expression using data from 182 PDAC patients in the TCGA database. ∗, *p* < 0.05. (E) mRNA expression levels of indicated genes in AsPC-1 cells with or without siRNA (*n* = 3 independent repeats). Data were expressed as mean ± SD. ∗, *p* < 0.05, ∗∗, *p* < 0.01, ∗∗∗, *p* < 0.001, ∗∗∗∗, *p* < 0.0001. (F) Colorimetric analysis of PKA activity in AsPC-1 cells transfected with siRNA targeting *EPHA2*, *SEMA4B*, *PYGB*, or *PRKAR2B* (*n* = 3 independent repeats). Data were expressed as mean ± SD. ∗∗, *p* < 0.01; NS, no significance. (G) Schematic diagram of PKA activation and inactivation. RS, regulatory subunit; CS, catalytic subunit. (H) Immunoblot analysis of PRKAR2B in H6C7, Capan-2, and AsPC-1 cells. (I) Immunoblot analysis of PRKAR2B in AsPC-1 cells with *PRKAR2B* knockout or overexpression. (J) Colorimetric analysis of PKA activity in AsPC-1 cells with *PRKAR2B* knockout or overexpression (*n* = 3 independent repeats). Data were expressed as mean ± SD. ∗, *p* < 0.05. (K) Immunoblot analysis of CREB, pCREB, and KLF5 in AsPC-1 cells with *PRKAR2B* knockout or overexpression. (L) Correlation between *PRKAR2B* and *KLF5* expression based on data from 182 PDAC patients in the TCGA database. ∗∗∗∗, *p* < 0.0001.

Although previous studies have confirmed PKA activation in PDAC, the underlying molecular mechanisms remain poorly understood. To address this deficiency in evidence, we first retrieved 501 human genes associated with the *activation of cAMP-dependent PKA pathway* from the PathCard database. Volcano plot analysis of transcriptomic data from the GSE226307 PDAC dataset revealed 41 significantly upregulated and 28 significantly downregulated genes within this pathway (Figure 2A). Among these 69 differentially expressed genes, 11 showed consistent expression changes in PDAC tissues based on TCGA data analysis (Figures 2B and S1C). Subsequent interrogation of the CPTAC proteomics database indicated that the protein expression levels of chloride intracellular channel 3 (CLIC3), EPH receptor A2 (EPHA2), macrophage stimulating 1 receptor (MST1R), semaphorin 4B(SEMA4B), glycogen phosphorylase B(PYGB), and PRKAR2B were altered in PDAC, in a manner consistent with their transcriptional changes (Figures 2C and S1D). Survival analysis using TCGA datasets further demonstrated that high expressions of *EPHA2*, *PYGB*, and *SEMA4B* were significantly associated with poorer overall survival in patients with PDAC (Figure S2A), while high expression of *PRKAR2B* was significantly associated with a high overall survival rate in patients with PDAC (Figure 2D).

*PRKAR2B* encodes the type IIβ regulatory subunit of PKA, a core component of the PKA holoenzyme that directly constrains its catalytic activity.^23^ In contrast, EPHA2, PYGB, and SEMA4B primarily function as downstream effectors or indirect modulators within the PKA signaling network.^24^^,^^25^^,^^26^ To assess the functional roles of these four genes in regulating PKA activity in PDAC, we performed siRNA-mediated knockdown of each gene in PDAC cell lines (Figure 2E). Subsequent colorimetric PKA activity assays showed that knockdown of *EPHA2* or *SEMA4B* resulted in a modest reduction in PKA activity, although these changes were not statistically significant (Figure 2F). In contrast, knockdown of *PRKAR2B* significantly increased PKA activity (Figure 2F). In addition, analysis of PDAC samples and control samples obtained from the GEO database (GSE226307 and GSE16515) demonstrated significant inhibition of *PRKAR2B* transcript levels (Figures 1A and 1C). These results suggest that downregulation of PRKAR2B may represent a key mechanism contributing to PKA activation in PDAC.

The inactive PKA holoenzyme is a tetramer consisting of two regulatory and two catalytic subunits^27^ (Figure 2G). Binding of cAMP to the regulatory subunits induces a conformational change that promotes dissociation of the holoenzyme, thereby releasing the active catalytic subunits (Figure 2G). Previous studies have reported transcriptional downregulation of *PRKAR2B* in PDAC, suggesting that it may contribute to PKA activation. However, the involvement of other PKA subunits in PDAC remains unclear. Analysis of RNA-sequencing data from PDAC tissues (GSE226307) revealed significant downregulation only in PRKAR2B expression (Figure 2A). No significant transcriptional changes were observed in other regulatory *(PRKAR1A, PRKAR1B,* and *PRKAR2A*) or catalytic subunit genes (*PRKACA* and *PRKACB*) (Figure 2A). Consistent with these findings, analysis of TCGA data confirmed that transcriptional alterations were specific to *PRKAR2B*, with no significant changes detected in the other subunits (Figure S2B). Furthermore, the protein expression level of PRKAR2B was inversely correlated with PKA activity in H6C7, Capan-2, and AsPC-1 cells (Figure 2H). Thus, PKA activation in PDAC appears to be specifically mediated by downregulation of PRKAR2B rather than alterations in other PKA subunits.

To further investigate the regulation of PKA activity by PRKAR2B, we generated *PRKAR2B* knockout and overexpression models in AsPC-1 cells (Figure 2I). Consistent with previous observations, colorimetric assays showed that PKA activity was enhanced by *PRKAR2B* knockout, and reduced by PRKAR2B overexpression^28^ (Figure 2J). Immunoblotting analysis further confirmed the results that *PRKAR2B* knockout enhanced phosphorylation of CREB, a direct readout of PKA activation, and increased protein levels of the downstream target KLF5 (Figure 2K). Conversely, PRKAR2B overexpression suppressed both CREB phosphorylation and KLF5 expression (Figure 2K). Analysis of TCGA data also revealed a significant negative correlation between *PRKAR2B* and *KLF5* transcript levels in PDAC tissues (Figure 2L). Summarily, these findings indicate that transcriptional downregulation of PRKAR2B is a key mechanism driving PKA activation in PDAC.

### Downregulation of transcription factor HHEX mediates transcriptional suppression of PRKAR2B

**Figure 3:**
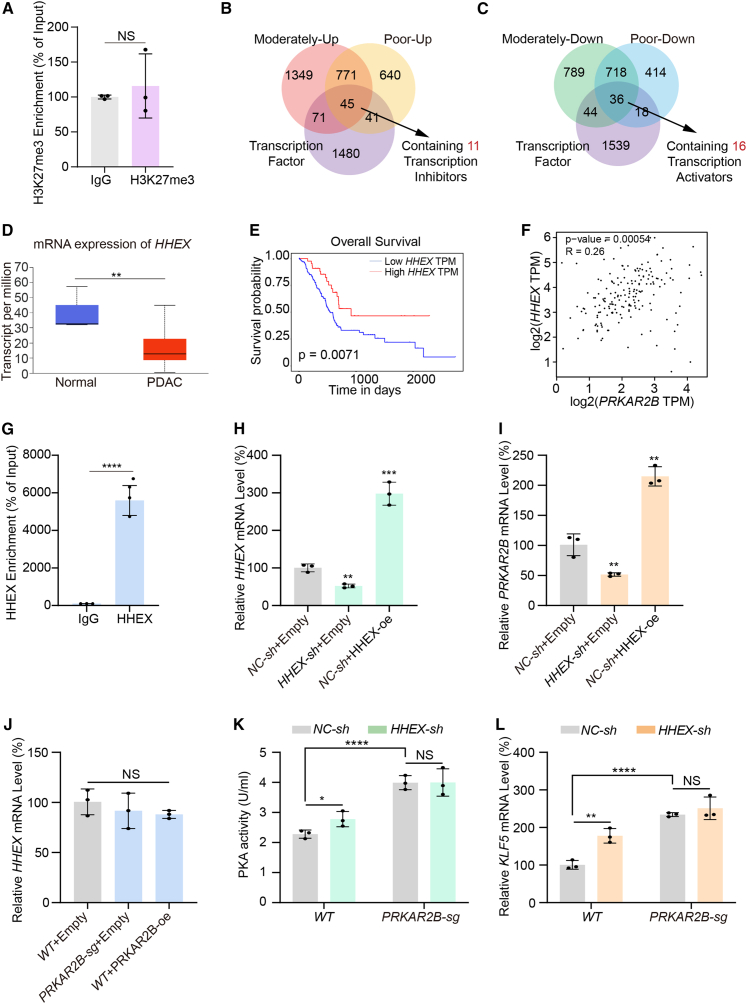
*HHEX* downregulation represses *PRKAR2B* transcription (A) Enrichment of H3K27me3 at the *PRKAR2B* gene in AsPC-1 cells. Chromatin immunoprecipitation was performed using an antibody against H3K27me3 or a control IgG antibody. The enriched DNA was quantified by qPCR and is presented as % of Input. Values are shown as mean ± SD (*n* = 3 independent repeats). NS, no significance. (B and C) Overlap of the upregulated (B) and downregulated (C) genes in moderate and poor differentiation PDAC compared with normal pancreatic tissue with known transcription factor. (D) mRNA expression profile of *HHEX* using 182 PDAC patient data and 4 normal controls from TCGA database. ∗∗, *p* < 0.01. (E) Kaplan-Meier survival analysis of *HHEX* expression using data from 182 PDAC patients in the TCGA database. ∗∗, *p* < 0.01. (F) Correlation between *PRKAR2B* and *HHEX* expression based on data from 182 PDAC patients in the TCGA database. ∗∗∗, *p* < 0.001. (G) Enrichment of HHEX at the *PRKAR2B* gene in AsPC-1 cells. Chromatin immunoprecipitation was performed using an antibody against HHEX or a control IgG antibody. The enriched DNA was quantified by qPCR and is presented as % of input. Values are shown as mean ± SD (*n* = 3 independent repeats). ∗∗∗∗, *p* < 0.0001. (H and I) mRNA levels of *HHEX* (H) or *PRKAR2B*(I) in AsPC-1 cells with *HHEX* knockdown or overexpression (*n* = 3 independent repeats). Data were expressed as mean ± SD. ∗∗, *p* < 0.01, ∗∗∗, *p* < 0.001. (J) mRNA levels of *HHEX* in AsPC-1 cells with *PRKAR2B* knockout or overexpression (*n* = 3 independent repeats). Data were expressed as mean ± SD. NS, no significance. (K–L) Colorimetric analysis of PKA activity (K) and qPCR analysis of *KLF5* mRNA levels (L) in AsPC-1 cells with or without *PRKAR2B* knockout followed by *HHEX* knockdown (*n* = 3 independent repeats). Data were expressed as mean ± SD. ∗, *p* < 0.05, ∗∗, *p* < 0.01, ∗∗∗∗, *p* < 0.0001; NS, no significance.

Our previous results established that transcriptional repression of PRKAR2B contributes significantly to aberrant PKA activation in PDAC. However, the mechanism underlying this repression remains unknown. Given the importance of epigenetic modifications in transcriptional control,^29^ and considering the role of the repressive histone mark Tri-methylation of histone H3 at lysine 27 (H3K27me3) in silencing developmental and identity-maintaining genes,^30^ we investigated whether H3K27me3 contributes to PRKAR2B repression in PDAC. We performed chromatin immunoprecipitation followed by quantitative PCR (ChIP-qPCR) using an *anti*-H3K27me3 antibody, to assess its enrichment at the promoter and putative regulatory regions of the *PRKAR2B* locus in AsPC-1 cells. We found no significant H3K27me3 enrichment at these regions (Figure 3A), indicating that H3K27me3-mediated repression is not the primary mechanism responsible for PRKAR2B downregulation in PDAC.

Subsequently, to examine whether dysregulated transcription factors contribute to the transcriptional repression of *PRKAR2B*, we intersected transcriptomic data from PDAC tissues (GSE226307) with transcription factor gene sets from the PathCard database. This analysis identified 11 upregulated transcriptional repressors and 16 downregulated transcriptional activators (Figures 3B and 3C). To validate the roles of these candidates, we analyzed PDAC samples from the TCGA database and found that the mRNA expression levels of *HHEX*, S*IM bHLH transcription factor 2 (SIM2)*, and *inhibitor of DNA binding 1 (ID1)* were consistent with our transcriptomic results (Figures 3D and S2C).

HHEX is a transcription factor with critical functions in the development of multiple organs, whose dysregulation has been frequently observed in hematologic malignancies and solid tumors.^31^ SIM2, a member of the bHLH-PAS transcription factor family, reportedly exhibits aberrant expression in several cancers, including those of the prostate and pancreas.^32^ ID1, an inhibitor of DNA binding, contributes to the pathogenesis and progression of various tumors, and elevated ID1 expression is often correlated with poor clinical prognosis.^33^ To further assess the potential involvement of these transcription factors in PRKAR2B repression in PDAC, we evaluated their association with patient survival. Only *HHEX* expression showed a significant correlation with overall survival (Figures 3E and S2D), suggesting its critical role in PDAC progression. Moreover, analysis of TCGA data revealed a significant positive correlation between *HHEX* and *PRKAR2B* mRNA levels (Figure 3F). ChIP-qPCR confirmed strong enrichment of HHEX at the *PRKAR2B* promoter (Figure 3G). Together, these results identified HHEX as a key transcription factor positively regulating *PRKAR2B* transcription in PDAC.

To further validate the transcriptional regulation of *PRKAR2B* by HHEX, we generated stable *HHEX* knockdown and overexpression models in AsPC-1 cells (Figure 3H). qPCR analysis showed that *HHEX* knockdown significantly reduced *PRKAR2B* mRNA levels, whereas HHEX overexpression markedly increased it, indicating that HHEX acts as a transcriptional activator of PRKAR2B (Figure 3I). Next, we examined whether PRKAR2B influences HHEX expression and found that modulation of PRKAR2B did not affect *HHEX* mRNA levels (Figure 3J). PKA activity assays revealed that knockdown of either *PRKAR2B* or *HHEX* enhanced PKA activity (Figure 3K). Moreover, *PRKAR2B* knockdown abolished the suppressive effect of HHEX overexpression on PKA activation (Figure 3K). Measurement of *KLF5* transcript levels further supported these findings (Figure 3L). Overall, these results demonstrate that HHEX downregulation suppresses *PRKAR2B* transcription, and that HHEX functions as a transcription factor for *PRKAR2B*, representing a key upstream mechanism driving PKA activation in PDAC cells.

### PKA activation drives malignant progression of PDAC through glucose metabolic reprogramming

**Figure 4:**
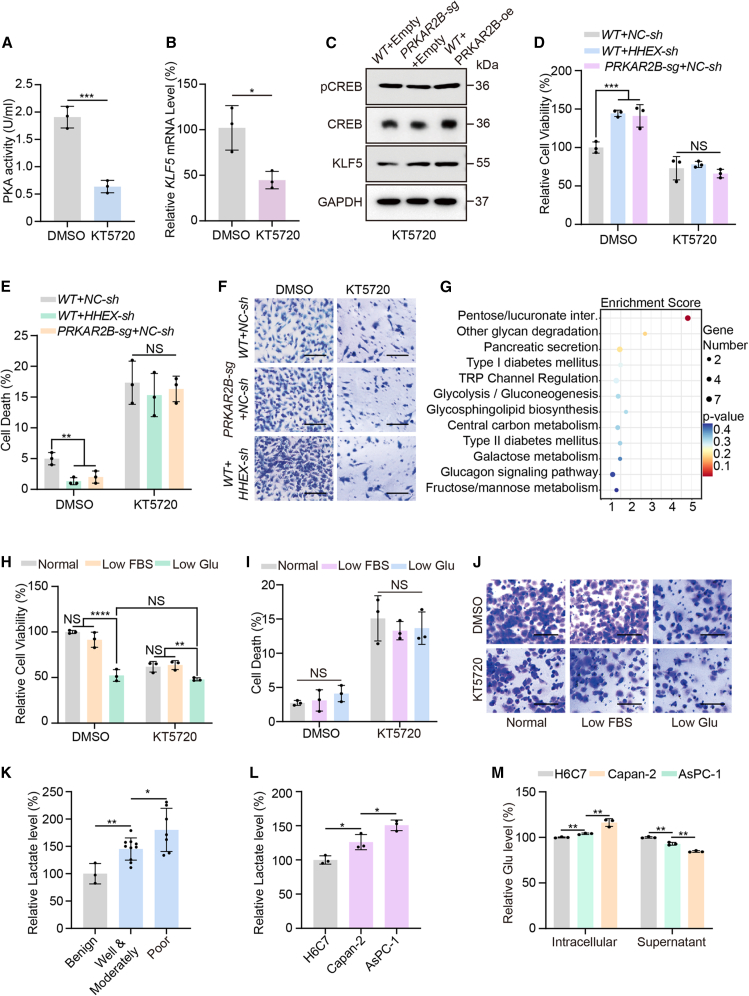
PKA activation promotes PDAC progression through metabolic reprogramming (A and B) Colorimetric analysis of PKA activity (A) and qPCR analysis of *KLF5* mRNA levels (B) in AsPC-1 cells treated with or without KT5720 (5 μM, 48 h) (*n* = 3 independent repeats). Data were expressed as mean ± SD. ∗, *p* < 0.05, ∗∗∗, *p* < 0.001. (C) Immunoblot analysis of CREB, pCREB, and KLF5 in AsPC-1 cells following *PRKAR2B* knockout or overexpression, with or without KT5720 treatment (5 μM, 48 h). (D) Cell viability was measured by CCK-8 assay in AsPC-1 cells subjected to *PRKAR2B* knockout or *HHEX* knockdown, in the presence or absence of treatment with KT5720 (5 μM, 48 h) (*n* = 3 independent repeats). Data were expressed as mean ± SD. ∗∗∗, *p* < 0.001; NS, no significance. (E) Cell death analysis by PI staining and flow cytometry in AsPC-1 cells subjected to *PRKAR2B* knockout or *HHEX* knockdown, in the presence or absence of treatment with KT5720 (5 μM, 48 h) (*n* = 3 independent repeats). Data were expressed as mean ± SD. ∗∗, *p* < 0.01; NS, no significance. (F) Cell invasion assays in AsPC-1 cells following *PRKAR2B* knockout or *HHEX* knockdown, with or without KT5720 treatment (5 μM, 48 h). Scale bars, 80 μm (G) KEGG pathway enrichment analysis of differentially expressed genes identified in GEO database (GSE134618). (H) Cell viability measured by CCK-8 assay in AsPC-1 cells under low FBS (2%) or low glucose (1,000 mg/L) conditions, with or without KT5720 treatment (5 μM, 48 h, *n* = 3 independent repeats). Data were expressed as mean ± SD. ∗∗, *p* < 0.01; ∗∗∗∗, *p* < 0.0001; NS, no significance. (I) Cell death analysis by PI staining and flow cytometry in AsPC-1 cells under low FBS (2%) or low glucose (1,000 mg/L) conditions, with or without KT5720 treatment (5 μM, 48 h, *n* = 3 independent repeats). Data were expressed as mean ± SD. NS, no significance. (J) Cell invasion assays in AsPC-1 cells treated with low FBS (2%), low glucose (1,000 mg/L), or KT5720 (5 μM, 48 h). Scale bars, 80 μm (K) Relative lactate levels in pancreatic puncture fluid from patients with benign pancreatic diseases (*n* = 3 independent samples), well or moderately differentiated PDAC (*n* = 10 independent samples), or poorly differentiated PDAC (*n* = 7 independent samples). Data were expressed as mean ± SD. ∗, *p* < 0.05; ∗∗, *p* < 0.01. (L) Relative lactate levels in the supernatant of H6C7, Capan-2, and AsPC-1 cells (*n* = 3 independent repeats). Data were expressed as mean ± SD. ∗, *p* < 0.05. (M) Intracellular and extracellular glucose levels in H6C7, Capan-2, and AsPC-1 cells (*n* = 3 independent repeats). Data were expressed as mean ± SD. ∗∗, *p* < 0.01.

Previous studies have shown that downregulation of the transcription factor HHEX in PDAC leads to reduced *PRKAR2B* transcription, thereby attenuating inhibition of the PKA catalytic subunits. However, the mechanisms through which PKA activation promotes PDAC malignancy remain poorly elucidated. To investigate this, we treated cells with the PKA inhibitor KT5720, which significantly reduced PKA activity and downregulated the expression of the PKA target gene *KLF5* (Figures 4A and 4B). Immunoblot analysis showed that KT5720 rescued the changes in CREB phosphorylation and KLF5 protein levels induced by modulation of PRKAR2B (Figure 4C). In contrast, KT5720 did not affect *PRKAR2B* transcription (Figure S2E). Collectively, these results indicate that KT5720 effectively suppresses PKA activity and downstream signaling without altering PRKAR2B expression.

Building on the effective suppression of PKA activity by KT5720, we subsequently evaluated the functional role of PKA in the malignant behavior of PDAC. Although PKA activation has been linked to proliferation, apoptosis, and metastasis in multiple cancer types, its specific contributions to PDAC are unclear. Our results showed that knockdown of *HHEX* or *PRKAR2B* significantly increased cell viability (Figure 4D), decreased cell death (Figure 4E), and enhanced cell invasion and migration (Figures 4F and S2F). Importantly, treatment with KT5720 abolished these phenotypic changes in proliferation, apoptosis, invasion, and migration, irrespective of whether they were induced by *HHEX* knockdown or *PRKAR2B* knockout (Figures 4D, 4E, and S2F). These findings indicate that PKA activation promotes malignant progression in PDAC by stimulating proliferation and migration, while inhibiting cell death.

Although the above-mentioned experiments demonstrated that PKA activation promotes malignant phenotypes in PDAC, the underlying molecular mechanisms remain incompletely understood. Therefore, we analyzed transcriptomic data (GSE134618) from human pluripotent stem cells treated with a PKA activator, identifying 1,415 differentially expressed genes (Figure S2G). KEGG pathway enrichment analysis of these genes revealed significant enrichment in 40 signaling pathways, including those closely associated with pancreatic function, such as glucose metabolism and insulin secretion (Figure 4G). These findings further validate the correlation between PKA activity and pancreatic function in an independent model, suggesting that metabolic reprogramming may represent one of the potential mechanisms through which the PKA signaling pathway promotes malignant tumor development. Given the central metabolic role of the pancreas and the established contribution of metabolic reprogramming to cancer progression,^34^^,^^35^ we hypothesized that PKA activation promotes PDAC metastasis by driving specific metabolic alterations.

To test this hypothesis, we established culture conditions mimicking distinct nutrient environments: AsPC-1 cells were maintained in normal medium (4,500 mg/L of glucose, 10% fetal bovine serum [FBS]), low-serum medium (4,500 mg/L of glucose, 2% FBS), or low-glucose medium (1,000 mg/L of glucose, 10% FBS), with all media supplemented with mannitol to maintain osmolarity. Cell proliferation assays showed that low-glucose conditions significantly inhibited proliferation, whereas the low-serum group showed no significant difference relative to the control (Figure 4H). Cell death assays indicated that neither low glucose nor low serum concentration significantly affected cell mortality (Figure 4I). Migration and invasion assays revealed that glucose deficiency markedly suppressed both processes (Figures 4J, S2H, and S3A). Notably, low-glucose treatment attenuated the inhibitory effects of the PKA inhibitor KT5720 on proliferation and metastasis (Figures 4H, 4J, S2H, and S3A). Conversely, KT5720 did not reverse the suppression caused by low glucose (Figures 4H, 4J, and S2H). Further analysis of patients with PDAC with varying differentiation grades revealed that the level of lactate, a key intermediate in glucose metabolism, in pancreatic puncture fluid was significantly higher in patients with poorly differentiated tumors than in those with moderate to well-differentiated tumors (Figure 4K). Both patient groups exhibited higher lactate levels than those with benign pancreatic diseases, who showed the lowest PKA activity (Figure 4K). A similar trend was observed across three cell lines with different PKA activity levels: as PKA activity increased, both lactate production and glucose utilization efficiency were elevated (Figures 4L–4M). Collectively, these results demonstrate that PKA-mediated promotion of PDAC cell proliferation and metastasis depends on glucose availability.

### Glucose-activated PKA drives HK2-mediated glycolysis in PDAC progression

Clinical and epidemiological evidence strongly links hyperglycemia with an elevated risk of PDAC,^36^ supported by the high comorbidity of diabetes in patients with PDAC and the frequent occurrence of new-onset type 2 diabetes as an early clinical indicator of the disease.^37^ Our experimental data further reinforced glucose dependence of PKA-driven proliferation and metastasis in PDAC. However, the precise molecular mechanisms by which glucose metabolism modulates PKA activity, and how PKA activation in turn reprograms glucose metabolism to facilitate malignant progression, remain to be fully elucidated.

**Figure 5:**
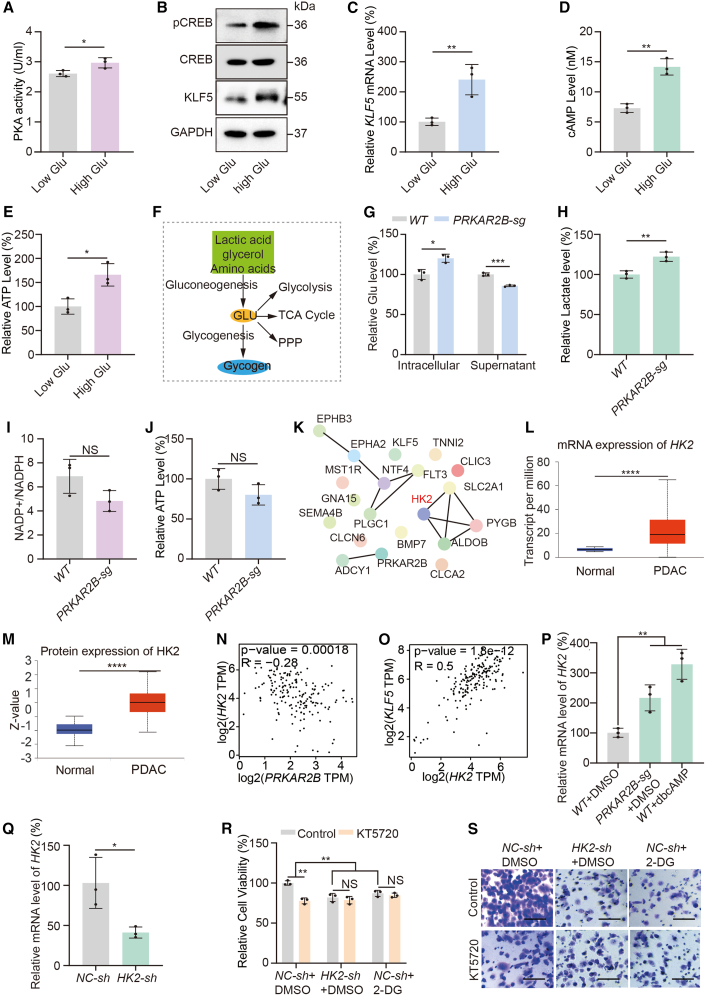
Glucose-activated PKA promotes PDAC progression via HK2-driven glycolysis (A) Colorimetric analysis of PKA activity in AsPC-1 cells treated with high glucose (4,500 mg/L) or low glucose (1,000 mg/L) (*n* = 3 independent repeats). Data were expressed as mean ± SD. ∗, *p* < 0.05. (B) Immunoblot analysis of CREB, pCREB, and KLF5 in AsPC-1 cells treated with high glucose (4,500 mg/L) or low glucose (1,000 mg/L). (C–E) mRNA expression of *KLF5* (C), intracellular cAMP levels (D), and relative ATP levels (E) in AsPC-1 cells treated with high glucose (4,500 mg/L) or low glucose (1,000 mg/L) (*n* = 3 independent repeats). Data were expressed as mean ± SD. ∗, *p* < 0.05, ∗∗, *p* < 0.01. (F) Schematic diagram of carbohydrate metabolism. TCA, tricarboxylic acid; PPP, pentose phosphate pathway. (G) Intracellular and extracellular glucose levels in AsPC-1 cells with or without *PRKAR2B* knockout (*n* = 3 independent repeats). Data were expressed as mean ± SD. ∗, *p* < 0.05, ∗∗∗, *p* < 0.001. (H) Relative lactate levels in the supernatant of AsPC-1 cultures with or without *PRKAR2B* knockout (*n* = 3 independent repeats). Data were expressed as mean ± SD. ∗∗, *p* < 0.01. (I–J) NADP^+^/NADPH ratio (I) and relative ATP levels (J) in AsPC-1 cells with or without *PRKAR2B* knockout (*n* = 3 independent repeats). Data were expressed as mean ± SD. NS, no significance. (K) Schematic diagram of the 19 core genes identified in the network analysis of Figure S3C. (L) mRNA expression levels of *HK2* based on data from 182 PDAC patients and 4 normal controls in the TCGA database. ∗∗∗∗, *p* < 0.0001. (M) Protein expression levels of HK2 based on data from 137 PDAC patients and 74 normal controls in the CPTAC database. ∗∗∗∗, *p* < 0.0001. (N–O) Correlation analysis among *HK2*, *KLF5*, and *PRKAR2B* expression using transcriptomic data from 182 PDAC patients in TCGA. ∗∗∗, *p* < 0.001, ∗∗∗∗, *p* < 0.0001. (P) *HK2* mRNA expression in AsPC-1 cells following *PRKAR2B* knockout or treatment with dbcAMP (0.2 mM, 24 h) (*n* = 3 independent repeats). Data were expressed as mean ± SD. ∗∗, *p* < 0.01. (Q) *HK2* mRNA expression in AsPC-1 cells with or without *HK2* knockdown (*n* = 3 independent repeats). Data were expressed as mean ± SD. ∗, *p* < 0.05. (R) Cell viability measured by CCK-8 assay in AsPC-1 cells after *HK2* knockdown or treatment with 2-DG (4 mM, 24 h) (*n* = 3 independent repeats). Data were expressed as mean ± SD. ∗∗, *p* < 0.01; NS, no significance. (S) Cell invasion assays in AsPC-1 cells after *HK2* knockdown or treatment with 2-DG (4 mM, 24 h). Data were expressed as mean ± SD. Scale bars, 80 μm.

To examine the regulatory effect of glucose on PKA activity in PDAC cells, we used the AsPC-1 cell line and measured PKA activation under different glucose conditions. The results showed that higher glucose concentrations significantly increased PKA activity (Figure 5A). Immunoblotting confirmed that high glucose markedly enhanced pCREB, a direct PKA substrate, and elevated protein levels of KLF5, a transcriptional target of PKA (Figure 5B). Concurrently, qPCR analysis indicated increased *KLF5* mRNA expression (Figure 5C). Together, these data indicate that a high-glucose environment activates the PKA signaling pathway. To explore the mechanism, we detected a pronounced rise in intracellular cAMP, the canonical activator of PKA, under high glucose conditions (Figure 5D). Since cAMP is produced by adenylate cyclase from ATP,^38^ we assessed cellular energy status and observed that high glucose substantially increased intracellular ATP levels (Figure 5E). In summary, high glucose promotes ATP production, which in turn enhances cAMP synthesis by adenylate cyclase, leading to activation of the PKA pathway.

Glucose metabolism represents a central hub for cellular energy production, biosynthesis, and the maintenance of metabolic homeostasis.^38^ Its major branches include glucose uptake, glycolysis, the pentose phosphate pathway (PPP), the tricarboxylic acid (TCA) cycle, and gluconeogenesis (Figure 5F). To determine how PKA activation influences glucose metabolism, we used a *PRKAR2B* knockout AsPC-1 cell model to constitutively activate PKA (Figure 2I). Measurement of intra- and extra-cellular glucose levels indicated that PKA activation significantly increased glucose uptake (Figure 5G). Consistent with enhanced glycolytic flux,^39^ lactate accumulation was markedly elevated in PKA-activated cells (Figure 5H). In contrast, PKA activation did not significantly alter the NADPH/NADP^+^ ratio, an indicator of oxidative PPP activity,^40^ or cellular ATP levels, which reflect integrated TCA cycle and oxidative phosphorylation activity^41^ (Figures 5I and 5J). These results demonstrate that PKA activation selectively promotes glycolysis without broadly affecting other major glucose metabolic pathways.

Although previous studies have shown that high glucose activates PKA and thereby promotes glycolysis in PDAC, the precise molecular mechanism through which PKA enhances glycolysis remains unclear. The PKA/CREB signaling pathway regulates multiple downstream targets. To systematically elucidate the manner in which PKA enhances glycolysis, we collected 501 core genes of the activation of cAMP-dependent PKA pathway and 46 core glycolysis genes from the PathCard database. By integrating these genes with a differentially expressed gene set from patients with poorly differentiated pancreatic cancer, we identified 69 PKA/CREB-related genes and ten glycolysis-related genes that were differentially expressed in poorly differentiated pancreatic cancer (Figure S3B). Further analysis of TCGA data revealed that 19 of these 79 genes showed statistically significant differential expression in pancreatic cancer tissues (Figure S3C). Subsequent protein-protein interaction network analysis of these 19 genes using the STRING database identified HK2 as a central regulatory node (Figure 5K). Data from the TCGA and CPTAC databases demonstrated that HK2 expression is significantly upregulated in PDAC samples (Figures 5L and 5M). HK2 is a mitochondrial outer membrane-bound rate-limiting glycolytic enzyme, which is predominantly expressed in insulin-sensitive tissues and overexpressed in tumors to drive the Warburg effect.^42^ As a central signaling node, PKA orchestrates transcriptional programs via direct phosphorylation of transcription factors,^43^ recruitment of epigenetic modifiers,^44^ and modulation of non-coding RNAs.^45^ Therefore, we hypothesized that PKA regulates glycolysis by controlling the transcription of *HK2*. Further analysis of TCGA datasets revealed a significant negative correlation between HK2 and PRKAR2B expression (Figure 5N), and a positive correlation between *HK2* and the PKA target gene *KLF5* (Figure 5O), suggesting that PKA activation may stimulate *HK2* transcription. We validated this experimentally by showing that both *PRKAR2B* knockout-mediated PKA activation and treatment with the PKA agonist dbcAMP significantly increased *HK2* mRNA levels (Figure 5P). These results indicate that PKA activation enhances glycolytic flux in PDAC cells, at least in part, through transcriptional upregulation of HK2.

Glycolysis provides not only rapid energy production and biosynthetic precursors but also contributes to an acidic tumor microenvironment through lactate secretion, thereby facilitating tumor proliferation and metastasis.^46^ To assess the functional contribution of glycolysis to malignant behaviors, we treated PDAC cells with 2-deoxy-D-glucose (2-DG), a specific glycolysis inhibitor, or knocked down the key glycolytic enzyme *HK2* in pancreatic cancer (Figure 5Q) to suppress glycolysis.^47^ Evaluation of the proliferation and invasion of AsPC-1 cells revealed that both HK2 knockdown and 2-DG treatment significantly inhibited AsPC-1 cell proliferation and invasion (Figures 5R and 5S). Notably, both 2-DG treatment and *HK2* knockdown abrogated the anti-proliferative and anti-metastatic effects of the PKA inhibitor KT5720 (Figures 5R and 5S). These results indicate that high glucose activates the PKA pathway, which upregulates HK2 expression and enhances glycolysis, thereby promoting proliferation and metastasis in PDAC.

### Glucose-activated PKA promotes glycolysis-dependent proliferation and metastasis

**Figure 6:**
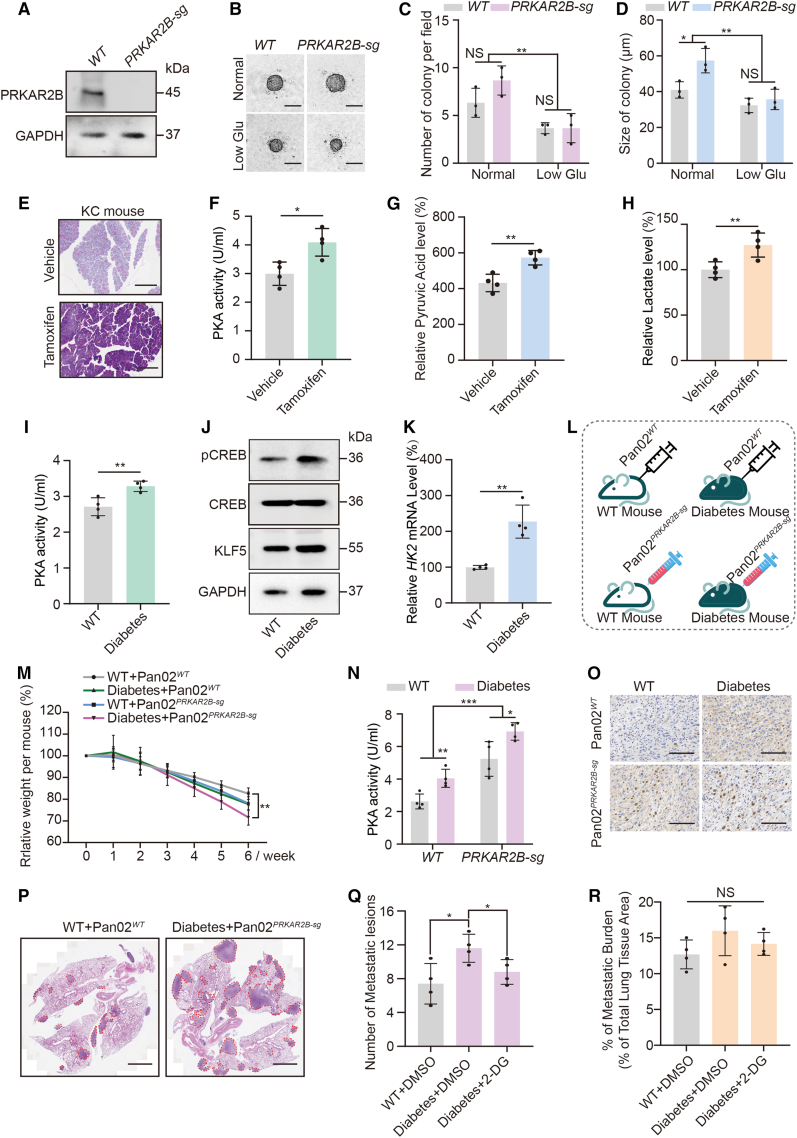
*In vivo* demonstration of glucose-activated PKA promoting glycolysis-dependent progression A. Immunoblot analysis of PRKAR2B in KPC cells with or without *PRKAR2B* knockout. (B–D) Representative images of colony formation by KPC cells, with or without *PRKAR2B* knockout, cultured in normal or low-glucose medium (B). Colony size (C) and number of colonies (D) were graphed (*n* = 3 independent repeats). Data were expressed as mean ± SD. Scale bars, 50 μm ∗, *p* < 0.05, ∗∗, *p* < 0.01, NS, no significance. (E) Representative H&E-stained pancreatic sections from mice with tamoxifen-inducible, pancreas-specific KRAS expression, harvested 3 weeks after tamoxifen or vehicle injection. Scale bars, 1 mm (F–H) PKA activity (F), pyruvic acid level (G), and lactate level (H) in pancreatic tissues from mice, harvested 3 weeks after tamoxifen or vehicle injection, assessing the effect of tamoxifen-inducible, pancreas-specific KRAS expression (*n* = 4 independent samples). Data were expressed as mean ± SD. ∗, *p* < 0.05; ∗∗, *p* < 0.01. (I) Colorimetric analysis of PKA activity in wild-type and diabetic mice (*n* = 4 independent samples). Data were expressed as mean ± SD. ∗∗, *p* < 0.01. (J) Representative immunoblot of CREB, pCREB, and KLF5 in wild-type and diabetic mice (*n* = 4 independent samples; see Figure S3F for all replicates). (K) mRNA expression level of *HK2* in wild-type and diabetic mice (*n* = 4 independent samples). Data were expressed as mean ± SD. ∗∗, *p* < 0.01. (L) Schematic diagram of the experimental design in mice. Pan02 cells with or without *PRKAR2B* knockout was injected into wild type or diabetic mouse through the tail vein (*n* = 4 independent samples). (M) Changes in mouse body weight following tail vein injection of *PRKAR2B*-knockout or wild-type Pan02 cells (*n* = 4 independent samples). Data were expressed as mean ± SD. ∗∗, *p* < 0.01. (N) Colorimetric analysis of PKA activity in tumor allografts from wild-type and diabetic mice with or without *PRKAR2B* knockout (*n* = 4 independent samples). Data were expressed as mean ± SD. ∗, *p* < 0.05, ∗∗, *p* < 0.01. (O) Representative image of Ki67 IHC staining in Pan02-control and Pan02-*PRKAR2B*-sg allografts from wild-type or diabetic mice (*n* = 4 independent samples). Scale bars, 150 μm (P) Representative H&E-stained lung sections at the experimental endpoint. Scale bars, 3 mm (Q–R) Quantification of metastatic lesion number (Q) and total metastatic burden (R) at the experimental endpoint across the indicated treatment groups (*n* = 4 independent samples). Data were expressed as mean ± SD. ∗, *p* < 0.05; NS, no significance.

To further validate this mechanism *in vitro* and *in vivo*, we employed KPC cells to devise an *in vitro* model and established a PKA-activated cell line by knocking out *PRKAR2B* (Figure 6A). The colony formation assay revealed that *PRKAR2B* knockout resulted in an increase in colony number and size, whereas this difference was reversed under low glucose conditions (Figures 6B–6D). For *in vivo* validation, we utilized pancreatic-specific LSL-K-ras G12D-Cre mice, a model that enables sustained expression of oncogenic Kras in pancreatic tissue and develops spontaneous pancreatic tumors upon tamoxifen induction. Three weeks after tamoxifen injection, the pancreatic tissues were collected for analysis. Although the observation period was insufficient to detect tumor formation, the pancreatic tissue exhibited structural disorganization and characteristic pre-neoplastic lesions (Figure 6E). Analysis showed that compared with controls, *KRAS* mutant mice displayed significantly elevated PKA activity, pyruvate levels, and lactate levels in pancreatic tissues (Figures 6F–6H). These findings align with our previous results and are consistent with increased PKA activity and enhanced glycolysis during pancreatic cancer progression. However, glucose-6-phosphate levels showed no significant change (Figure S3D), which may be attributed to multifactorial regulation involving glucose transport, hexokinase activity, and allosteric activation of phosphofructokinase-1.^48^ We employed a diabetic mouse model to further investigate the impact of blood glucose levels on pancreatic cancer progression. Blood glucose levels were measured at 3-day intervals throughout the experimental period. Diabetic mice exhibited significantly elevated blood glucose levels compared with the wild-type controls (Figure S3E). Analysis of pancreatic tissues showed increased PKA activity, higher phosphorylation of the PKA substrate CREB, and elevated protein expression of the PKA target KLF5 in the diabetic group (Figures 6I, 6J, and S3F), indicating hyperactivation of the PKA pathway under hyperglycemic conditions. Moreover, qPCR analysis revealed increased HK2 transcription in pancreatic tissues retrieved from diabetic mice (Figure 6K). Collectively, these *in vivo* results support the mechanism whereby glucose promotes PKA activation and subsequent HK2 upregulation.

To evaluate the effect of glucose-mediated PKA activation on tumor proliferation and metastasis *in vivo*, *wild-type*-Pan02 or *PRKAR2B*-sg-Pan02 cells were intravenously injected into the tail veins of wild-type and diabetic mice, yielding four experimental groups (Figure 6L). The Pan02 cell line displays hybrid-mesenchymal features and high metastatic potential, largely driven by the PI3K/AKT-c-MET signaling axis, making it a suitable model for studying PDAC metastasis.^49^ At the sixth week, all mice were euthanized. Body weight analysis revealed that diabetic mice injected with *PRKAR2B*-knockout Pan02 cells via the tail vein experienced the most pronounced weight loss, demonstrating the highest tumor burden and a significant difference compared to *wild-type* Pan02 mice injected with *wild-type* cells (Figure 6M). Tumors derived from *PRKAR2B*-sg-Pan02 cells showed significantly higher PKA activity compared with those originating from *wild-type*-Pan02 cells (Figure 6N). Moreover, tumors in diabetic mice exhibited increased PKA activity relative to those in normoglycemic hosts (Figure 6N). Cell proliferation, which was assessed using Ki67 immunohistochemical staining, followed a similar pattern to PKA activity (Figures 6O and S3G). Hematoxylin and eosin staining was used to evaluate the metastatic burden in pancreatic, liver, and lung tissues, with the most notable differences observed in the lungs. Mice bearing *PRKAR2B*-sg-Pan02 cells under diabetic conditions developed the highest number of pulmonary metastatic foci and the largest area of pulmonary invasion (Figures 6P and S3H–S3I). In contrast, *wild-type* mice inoculated with *wild-type*-Pan02 cells showed the fewest metastatic foci and the smallest area of lung invasion (Figures 6P and S3H–S3I). The differences between these two groups were statistically significant (Figures S3H–S3I). Collectively, these *in vivo* results confirm the tumor-promoting role of PKA activation and highlight its critical contribution to proliferation and metastasis, particularly in a hyperglycemic microenvironment.

Given the important role of glycolysis in PKA-mediated malignant progression identified in our previous experiments, we evaluated the anti-metastatic effect of the glycolytic inhibitor 2-DG *in vivo*. Quantitative analysis showed that 2-DG treatment significantly reduced the number of pulmonary metastatic foci compared with the untreated control group, although it had little effect on the size or invasive area of established metastases (Figures 6Q–6R). These results suggest that inhibition of glycolysis effectively blocks the metastatic colonization of tumor cells but has limited impact on the growth or expansion of existing metastatic lesions.

## Discussion

This study systematically elucidated a novel metabolic-signaling axis that regulates the malignant progression of PDAC, revealing, for the first time, sustained activation of the PKA signaling pathway in this context. PKA activation results not only from direct stimulation by cAMP produced under hyperglycemic conditions, but also from transcriptional downregulation of the regulatory subunit *PRKAR2B* due to loss of the transcription factor HHEX, which alleviates suppression of the catalytic subunit. Activated PKA subsequently upregulates *HK2* transcription, enhances glycolytic flux, and ultimately promotes PDAC proliferation and metastasis. These findings advance our understanding of the molecular mechanisms underlying metabolic reprogramming in PDAC and provide new insights for developing precision therapeutic strategies.

This study focused on the cAMP signaling pathway, which was found to be significantly enriched in KEGG analysis, suggesting its potential role in PDAC progression. The analysis also highlighted other enriched pathways, including cGMP-PKG, NF-κB, and cytokine-cytokine receptor interaction. Previous studies have shown that the cGMP-PKG pathway can suppress EMT and induce cell-cycle arrest or apoptosis in certain contexts.^50^ However, in the presence of *KRAS* mutations or SMAD family member-4 loss, it may promote tumorigenesis through metabolic reprogramming or crosstalk with the mitogen activated kinase-like protein pathway.^51^ The NF-κB pathway, a known contributor to chemotherapy resistance, enhances gemcitabine resistance via 3-oxoacid CoA-transferase 1 upregulation and cooperates with mutant *KRAS* to facilitate invasion and metastasis.^52^ The cytokine-receptor network helps foster an immunosuppressive microenvironment, for example, tumor-associated Schwann cells secrete inhibitory ligands that impair CD8^+^ T cell function,^53^ and lysine demethylase 5B-mediated suppression of the STING pathway leads to reduced T cell infiltration,^54^ both of which contribute to resistance to immune checkpoint inhibitors. Although these pathways were not the central focus of our study, combination therapies targeting these mechanisms represent a promising direction for overcoming current therapeutic limitations and may hold significant clinical translational value.

In recent years, a large body of evidence on the regulatory interplay between metabolic and signaling pathways has been accumulated.^55^ Mounting evidence indicates that cells maintain metabolic homeostasis by integrating nutrient availability with signal transduction mechanisms.^56^ For example, G protein-coupled receptor signaling modulates processes such as glycolysis and fatty acid oxidation through the cAMP-PKA cascade in response to hormonal stimuli.^57^ Metabolites can also directly modify histones or transcription factors, thereby shaping transcriptional programs.^58^ This study unveiled the dual mechanism of PKA regulation in PDAC involving both transcriptional and metabolic inputs. On one hand, downregulation of the transcription factor HHEX leads to reduced expression of the PKA regulatory subunit PRKAR2B, resulting in increased liberation of catalytic subunits and elevated basal PKA activity. On the other hand, a high-glucose microenvironment enhances AC activity, increasing cAMP production and further activating PKA, a response experimentally validated in diabetic models. Elucidation of this cross-regulatory mechanism not only provides the first evidence of deep metabolic-signaling integration in PDAC but also establishes a conceptual framework for further mechanistic investigation.

The current clinical stratification of PDAC, which remains heavily reliant on imaging and conventional biomarkers such as CA19-9, is beset by limitations including interference from biliary obstruction and non-expression in Lewis antigen-negative individuals (5%–10% of the population).^59^^,^^60^^,^^61^ While adjunctive markers (e.g., CEA, CA125, and CA242) may marginally improve sensitivity, their specificity and early diagnostic utility are suboptimal.^62^^,^^63^^,^^64^^,^^65^ In this context, our study reveals a significant inverse correlation between PKA activity and the PDAC differentiation grade, with elevated activity in poorly differentiated tissues and reduced activity in their well-differentiated counterparts. This finding suggests that PKA activity may serve as a functional indicator of tumor aggressiveness. Notably, we identified PRKAR2B, a regulatory subunit of PKA, as a potential dual-purpose target. Its inhibition in PDAC tissues correlates directly with PKA hyperactivity, enabling dynamic malignancy assessment via minimally invasive testing. More importantly, targeted inhibition of PRKAR2B attenuated metabolic dysregulation and suppressed tumor growth in preclinical models, implying therapeutic applicability. While these results position PRKAR2B as a promising biomarker and therapeutic candidate, several aspects require further validation. First, the mechanisms linking PRKAR2B to metabolic reprogramming need elucidation. Second, our findings are derived from tissue-based assays and validation in liquid biopsies is essential for clinical translation. Finally, while PRKAR2B inhibition shows efficacy, its specificity and off-target effects warrant investigation. Future studies should compare the diagnostic performance of PRKAR2B against existing biomarkers in larger cohorts and explore its synergy with conventional therapies.

In summary, this study proposes a metabolic-signaling axis linking hyperglycemia to sustained PKA activation, which upregulates HK2 expression and enhances glycolytic flux in PDAC. These findings suggest that PKA activity and PRKAR2B expression may serve as potential biomarkers for malignancy grading, particularly in identifying poorly differentiated subtypes.

### Limitations of the study

We acknowledge that the statistical power of our initial clinical correlation based on the 20-case Shanghai cohort was limited, especially for stratified analyses. However, subsequent validation using the TCGA-PAAD cohort confirmed the prognostic significance of our identified axis, bolstering the reliability of our conclusions. Furthermore, the direct association between hyperglycemia and PDAC progression highlights the clinical relevance of metabolic interventions. While this study provides a theoretical basis for synergistic strategies combining glycemic control with targeted therapies, several limitations warrant consideration. First, the mechanistic links between hyperglycemia and PKA activation require further *in vivo* validation. Second, the biomarker performance of PRKAR2B needs assessment in larger clinical cohorts, especially across diverse patient populations. Future studies should explore whether metabolic modulation can augment the efficacy of existing PDAC therapies and evaluate long-term outcomes in preclinical models.

## Resource availability

### Lead contact

Further information and requests for resources and reagents should be directed to and will be fulfilled by the lead contact, Lin Wang (18817314343@sjtu.edu.cn).

### Materials availability

This study did not generate new unique reagents.

### Data and code availability

• All data reported in this article will be shared by the lead contact upon request.
• This study did not generate new code.
• Any additional information required to reanalyze the data reported in this article is available from the lead contact upon reasonable request.

## Acknowledgments

This work was supported by grants from the 10.13039/100014718National Natural Science Foundation of China (82273418) and the project of 10.13039/501100018625Shanghai Science and Technology Commission (22Y11902800).

## Author contributions

Conceptualization, L.W.; methodology, J.W. and Q.L.; investigation, J.W. and Q.L.; data analysis, J.L., S.X., W.L., and F.W.; writing – original draft, L.W. and J.W.; writing – review & editing, L.W., J.L., and J.W.; supervision, L.W. and J.L.; funding acquisition, L.W. and J.L.; resources, L.W. and J.L.

## Declaration of interests

The authors declare no competing interests.

## STAR★Methods

### Key resources table

**Table:** 

REAGENT or RESOURCE	SOURCE	IDENTIFIER
**Antibodies**		

Ki67	Cell Signaling Technology	Cat#12202
GAPDH	Santa Cruz Biotechnology	Cat#sc-137179
PRKAR2B	Santa Cruz Biotechnology	Cat#sc-376778
CREB	Abcam	Cat#ab31515
pCREB	Abcam	Cat#ab32096
KLF5	Santa Cruz Biotechnology	Cat#sc-398014
HK2	Santa Cruz Biotechnology	Cat#sc-130358
H3K27me3	Cell Signaling Technology	Cat#9733
HRP-conjugated anti-rabbit IgG	Cell Signaling Technology	Cat#7074
HRP-conjugated anti-mouse IgG	Cell Signaling Technology	Cat#7076

**Chemicals, peptides, and recombinant proteins**		

Streptozotocin (STZ)	MCE	Cat#HY-13753
Tamoxifen	Sigma-Aldrich	Cat#579000
Propidium Iodide (PI)	Beyotime	Cat#C1352M
Crystal Violet	Beyotime	Cat#C0121
Matrigel	Corning	Cat#CLS354277

**Critical commercial assays**		

Lipofectamine 2000	Invitrogen	Cat#11668019
CCK-8 kit	Beyotime	Cat#C0038
PKA activity assay kit	Invitrogen	Cat#MAN0019010
KLF5 ELISA kit	Biomatik	Cat#EKU05520
phospho-CREB ELISA kit	R&D Systems	Cat#DYC2510-2
ChIP-IT Express Kit	Active Motif	Cat#53032
RNeasy Mini Kit	Qiagen	Cat#74104
iTaq Universal One-Step RT-qPCR Kit	Bio-Rad	Cat#1725150
Glucose Assay Kit	Beyotime	Cat#S0201S
L-Lactate Assay Kit	Beyotime	Cat#S0208S
CellTiter-Glo Luminescent Cell Viability Assay	Promega	Cat#G7570
cAMP Direct Immunoassay Kit	Abcam	Cat#ab138880
NADP+/NADPH Assay Kit	Beyotime	Cat#S0179
Amplex Red Pyruvic Acid Detection Kit	Beyotime	Cat#S0299S
Glucose-6-phosphate (G6P) Detection Kit	Beyotime	Cat#S0185

**Experimental models: Cell lines**		

Human: HPDE6-C7 (H6C7)	Cell Bank of Chinese Academy of Sciences	Cat#BFN60807571
Human: Capan-2	Cell Bank of Chinese Academy of Sciences	Cat#BFN60807521
Human: AsPC-1	Cell Bank of Chinese Academy of Sciences	Cat#BFN60700314
Mouse: KPC-Luc	Shanghai Model Organisms Center	Cat#NM-YD04-TG01
Human: HEK293T	Laboratory of Jiayi Wang (Shanghai Chest Hospital)	

**Experimental models: Organisms/strains**		

Mouse: C57BL/6	Shanghai Model Organisms Center	Strain code: C57BL/6Smoc; Cat#SM-001
Mouse: Pdx1-2A-CreERT2	Shanghai Model Organisms Center	Strain code: C57BL/6Smoc-Pdx1em1(Avi tag-2A-CreERT2)Smoc; Cat#NM-KI-18042
Mouse: LSL-K-rasG12D	Laboratory of Jiayi Wang (Shanghai Chest Hospital)	Strain code: C57BL/6Smoc-Trp53em4(R172H)Krasem4(LSL-G12D)Tg(Pdx1-cre)Smoc; Cat#NM-KI-210096

**Oligonucleotides**		

siRNA, shRNA and sgRNA	GentleGen	Table S2
PCR primers for qRT-PCR	GentleGen	Table S2

**Other**		

Patient characteristics and clinical data	This paper	Table S1

### Experimental model and study participant details

#### Human subjects

Pancreatic juice samples were collected via puncture from patients with benign pancreatic diseases or PDAC at Shanghai General Hospital. Relevant patient characteristics are summarized in Table S1. Written informed consent was obtained from all participants prior to sample collection. This study was conducted in accordance with the ethical principles of the Declaration of Helsinki and was approved by the Institutional Ethics Committee of Shanghai General Hospital(Approval number: 2024[094]). The influence of patient sex on the reported results was not analyzed in this study, as the sample size was not powered for such analysis.

#### Animals

Male C57BL/6 mice (6–8 weeks) and female Pdx1-2A-CreERT2 mice (a Cre-driver line for pancreatic specific gene manipulation, 6–8 weeks) were purchased from Shanghai Model Organisms Center. Male LSL-K-rasG12D mice (a conditional mutant allele for oncogenic Kras, 6–8 weeks) were generously provided by the laboratory of Jiayi Wang at Shanghai Chest Hospital. All mice were housed under specific pathogen-free conditions with a 12-h light/dark cycle. All animal procedures were approved by Shanghai First People’s Hospital Clinical Center Laboratory Animal Welfare & Ethics Committee(Approval number: 2024AWS005). Only male mice of the C57BL/6 strains were used in the metastasis experiments; therefore, the influence of sex was not assessed for these models. The female Pdx1-2A-CreERT2 mice were used solely for breeding purposes.

#### Cell lines

The human pancreatic cancer cell lines HPDE6-C7 (H6C7, cat. BFN60807571), Capan-2 (cat. BFN60807521), and AsPC-1 (cat. BFN60700314) were obtained from Cell Bank of Chinese Academy of Sciences (Shanghai, China). KPC-Luc cell line (cat. NO. NM-YD04-TG01) was purchased from Shanghai Model Organisms Center (Shanghai, China). The HEK293T cell line was kindly provided by the laboratory of Jiayi Wang at Shanghai Chest Hospital. H6C7 cells were maintained in Keratinocyte SFM medium (Cat. 17005042, Invitrogen, Jiangsu Province, China) supplemented with epidermal growth factor, bovine pituitary extract, and 1× antibiotic-antimycotic (Gibco, Cat. 15240-062). Capan-2 cells were cultured in McCoy’s 5A (M8403, Sigma, Shanghai, China) medium supplemented with 10% fetal bovine serum (FBS) and 1% penicillin-streptomycin (100 U/mL penicillin, 100 μg/mL streptomycin). AsPC-1 and Pan02 cells were grown in RPMI 1640 medium (R8758, Sigma) containing 10% FBS and 1% penicillin-streptomycin. HEK293T and KPC-Luc cells were cultured in Dulbecco’s Modified Eagle Medium (DMEM, Cat. SH30022.FS, HyClone) supplemented with 10% FBS and 1% penicillin-streptomycin. All cell lines were regularly tested for mycoplasma contamination and were authenticated using short tandem repeat profiling. None of the cell lines used in this study are listed in the ICLAC database of commonly misidentified cell lines.

#### Ethics statement

Human samples were collected with the approval of the Institutional Ethics Committee of Shanghai General Hospital(Approval number: 2024[094]). All animal protocols and procedures were approved by Shanghai First People’s Hospital Clinical Center Laboratory Animal Welfare & Ethics Committee(Approval number: 2024AWS005).

### Method details

#### Vector construction and cell transfection

HEK293T cells were transfected using Lipofectamine 2000 (Cat. 11668019, Invitrogen, MA, USA) according to the manufacturer’s instructions. siRNAs targeting *EPHA2, SEMA4B, PYGB*, and *PRKAR2B* were purchased from GenePharma (Shanghai, China). Lentiviral particles were generated by co-transfecting HEK293T cells with either pLKO- or pLX-based expression constructs (encoding PRKAR2B, or HHEX), shRNA constructs targeting *HHEX* or *HK2*, or sgRNA constructs targeting *PRKAR2B* along with standard packaging plasmids. All the plasmids were obtained from GentleGen (Jiangsu Province, China). Viral supernatants were harvested 48 h after transfection. Target cells were transduced in the presence of polybrene (8 μg/mL), and successfully transduced cells were selected with puromycin (1 μg/mL) for 4–5 days.

#### Animal experiments

C57BL/6 mice (8 weeks old) were used for *in vivo* studies. Diabetes was induced by intraperitoneal injection of streptozotocin (STZ, 50 mg/kg, cat.HY-13753, MCE, Shanghai, China) dissolved in 0.1 M sodium citrate buffer (pH 4.5) for five consecutive days. Control mice received injections of the citrate buffer vehicle alone. Two weeks after the first STZ injection, diabetes was confirmed by measuring blood glucose levels after a 6-h fast using Accu-Chek test strips (Roche, Basel, Switzerland). Mice with blood glucose levels exceeding 11 mmol/L were considered diabetic and included in subsequent experiments.

Pdx1-2A-CreERT2 mice were bred with LSL-K-rasG12D mice. Offspring carrying both alleles were identified by genotyping and used in subsequent experiments. At 6 weeks of age, mice were administered tamoxifen (Sigma-Aldrich, cat.579000, 100 mg/kg, dissolved in corn oil) or vehicle (corn oil) via intraperitoneal injection for five consecutive days. Three weeks after the final injection, the mice were euthanized, and pancreatic tissues were collected for H&E staining, PKA activity assay, and quantification of pyruvate, lactate, and G6P levels.

#### Lung metastasis analysis

For lung metastasis studies, C57BL/6 mice were injected intravenously via the tail vein with Pan02 cells (1 × 10^6^ cells in 100 μL of DPBS) transduced with either control or *PRKAR2B*-targeting sgRNA. Three weeks after injection, the mice were euthanized, and lungs were harvested for metastatic analysis. The lungs were fixed in 4% paraformaldehyde overnight, washed, and the number of surface metastatic lesions was counted using microscope. Subsequently, the tissue samples were processed for histology, embedded in paraffin, sectioned, and subjected to H&E staining. The metastatic burden was quantified by measuring the percentage of the metastatic area relative to the total lung area on histological sections using an Aperio eSlide Capture Device (Leica Biosystems).

#### Soft agar colony formation assay

The soft agar colony formation assay was performed in 6-well plates. First, a base layer comprising 1% agar in complete DMEM (supplemented with 10% FBS) was solidified. Subsequently, a top layer containing 0.6% agar in DMEM mixed with 5,000 KPC-Luc cells per well was overlaid. The cells with or without *PRKAR2B* knockout were then cultured in either standard DMEM or low-glucose cell culture medium (cat. C11885500CP, Gibco), both supplemented with 10% FBS and 1% penicillin–streptomycin. Colonies were imaged using a Zeiss Axio Observer microscope. Quantitative analysis included full-well colony counting, diameter measurement of three randomly selected colonies per group using ZEN 3.2 software, and acquisition of representative images. Each experimental condition was performed in three biological replicates.

#### Immunohistochemistry

Tissue sections were first deparaffinized and rehydrated through a graded ethanol series. Antigen retrieval was performed by heating the sections in 10 mM sodium citrate buffer (pH 6.0, cat. C1013, Solarbio, Jiangsu Province, China) for 30 min. Endogenous peroxidase activity was blocked by incubation in 3% hydrogen peroxide for 20 min at room temperature. The sections were then incubated in a blocking solution containing 5% BSA, 1% goat serum, and 0.1% Tween 20 in PBS for 1 h at room temperature. Primary antibody against Ki67 (1:500 dilution, cat. 12202, Cell Signaling Technology, MA, USA) was prepared in SignalStain Antibody Diluent (cat. 8112, Cell Signaling Technology) and applied to the sections overnight at 4°C. The following day, the slides were incubated with a biotin-conjugated secondary antibody for 1 h at room temperature. Signal amplification and detection were performed using the Vectastain Elite ABC kit (cat. PK-6105, Vector Laboratories, CA, USA), followed by development with diaminobenzidine (DAB). Hematoxylin was used for nuclear counterstaining. Finally, the sections were dehydrated, cleared, and mounted for microscopic evaluation.

#### Immunoblotting

Cells were lysed in ice-cold Western and IP lysis buffer (cat. P0013, Beyotime, Shanghai, China) for 30 min on ice. The lysates were then centrifuged at 12,000 × g for 15 min at 4°C, and the supernatants were collected for immunoblotting. Protein concentration was determined using a BCA protein assay kit (cat. P0012, Beyotime). The following primary antibodies were used: GAPDH (1:1000, cat. sc-137179, Santa Cruz Biotechnology), PRKAR2B (1:1000, cat. sc-376778, Santa Cruz Biotechnology), CREB (1:1000, cat. ab31515, Abcam), pCREB (1:1000, cat. ab32096, Abcam), KLF5 (1:1000, cat. sc-398014, Santa Cruz Biotechnology), and HK2 (1:1000, cat. sc-130358, Santa Cruz Biotechnology). Horseradish peroxidase (HRP)-conjugated anti-rabbit IgG (1:3000, cat. 7074, Cell Signaling Technology) and anti-mouse IgG (1:3000, cat. 7076, Cell Signaling Technology) were used as secondary antibodies.

#### Cell counting kit-8 assay

In the cell viability assay, 2000 cells were seeded per well in 96-well plates and allowed to adhere overnight. CCK-8 reagent (cat.c0038, Beyotime) was added to each well at a 1:10 dilution in fresh culture medium, and the plates were incubated for 1–4 h at 37°C. Absorbance was measured at 450 nm using a microplate reader. The experiment was performed with at least three technical replicates and repeated independently three times.

#### Flow cytometry

Cells were stained with 5 μg/mL propidium iodide (PI, cat. C1352M, Beyotime) for 15–30 min at room temperature in the dark. After washing with PBS to remove unbound dye, the cells were immediately analyzed using a BD FACSCanto II flow cytometer. Data acquisition was performed using BD FACSDiva software, and subsequent analysis was conducted with FlowJo software (v10.4).

#### Migration assay

Cell migration was assessed using a scratch wound healing assay. Briefly, 5 × 10^5^ cells were seeded per well in 6-well plates and cultured in DMEM supplemented with 10% FBS until they reached 90–100% confluence. The medium was then replaced with serum-free DMEM, and cells were serum-starved for 6 h. A uniform scratch was created in each well using a sterile 200 μL pipette tip. After washing with PBS to remove detached cells, fresh serum-free medium was added. Images of the scratch were captured at 0 and 24 h using an inverted microscope. The extent of migration was quantified by measuring the initial scratched area (S_0_) and the remaining area at each time point (S_t_) using ImageJ software. The percentage of wound closure was calculated as (S_0_ - S_t_)/S_0_ × 100%. Each experiment was performed in triplicate and repeated at least three times independently.

#### Transwell assay

Cell migration was further evaluated using a transwell assay. The lower chamber of a 24-well Transwell plate (cat. CLS3422, Corning, Shanghai, China) was filled with 500 μL of complete medium containing 10% FBS. A total of 5 × 10^4^ cells resuspended in 200 μL of serum-free medium were seeded into the upper chamber pre-coated with Matrigel (cat. CLS354277, Corning). After incubation for 24 h at 37°C, non-migratory cells on the upper surface were carefully removed with a cotton swab. Cells that had migrated to the lower surface were fixed with 4% paraformaldehyde for 30 min and stained with 0.1% crystal violet (cat. C0121, Beyotime) for 20 min. The stained cells were imaged and quantified by counting in five random fields per well under an inverted microscope. Each experiment was performed in triplicate and repeated at least three times.

#### PKA activity assay

PKA activity was measured using a commercial colorimetric activity assay kit (cat. MAN0019010, Invitrogen) according to the manufacturer’s instructions. Briefly, cells were lysed in the provided Activated Cell Lysis Buffer and incubated on ice for 30 min with intermittent vortexing. The lysates were centrifuged at 10,000 × g for 10 min at 4°C, and the supernatant was collected. Protein concentration was determined, and samples were appropriately diluted with 1× Kinase Reaction Buffer to ensure measurements fell within the linear range of detection. Then, 40 μL of each diluted sample and 10 μL of reconstituted ATP solution were added to the designated wells. The plate was sealed and incubated at 30°C for 90 min with gentle shaking. After incubation, each well was washed four times with 300 μL of wash buffer, and residual liquid was removed by gently tapping the plate on absorbent paper. Next, 25 μL of Phospho PKA Substrate Antibody was added to each well and incubated for 60 min at room temperature with shaking. Following another washing step, 25 μL of Donkey anti-Rabbit IgG HRP Conjugate was added and incubated for 60 min under the same conditions. After a final wash, 100 μL of TMB substrate solution was added to each well and incubated for 30 min at room temperature. The reaction was stopped by adding 50 μL of stop solution, and the absorbance was measured at 450 nm. PKA activity was calculated based on a standard curve generated using the provided standards and analyzed with curve-fitting software.

#### ELISA for KLF5

KLF5 protein levels were measured using a KLF5 ELISA kit (EKU05520, Biomatik, Kitchener, Canada). Pancreatic puncture samples were lysed and centrifuged, and 100 μL of the supernatant was added to the wells. Following incubation at 37°C for 1 h, the wells were washed. Then, 100 μL of Detection Reagent A was added, and the plate was incubated for 1 h at 37°C. After washing, 100 μL of Detection Reagent B was added and incubated for 30 min at 37°C. Following a wash step, 90 μL of substrate solution was added to each well and incubated for color development. The reaction was stopped, and the optical density was measured at 450 nm.

#### ELISA for pCREB

phospho-CREB levels were measured using a phospho-CREB ELISA kit (DYC2510–2, R&D Systems, Shanghai, China). A 96-well plate was coated with capture antibody (100 μL/well) and incubated overnight at room temperature. After blocking and washing, pancreatic puncture samples (100 μL/well) were added and incubated for 2 h at room temperature. Following another wash, 100 μL of detection antibody was added and incubated for 2 h. After washing, 100 μL of Streptavidin-HRP was added and incubated for 20 min. The plate was washed again, and 100 μL of substrate solution was added for color development. The reaction was terminated, and the absorbance was measured at 450 nm.

#### ChIP-qPCR

ChIP was performed using the ChIP-IT Express Kit (cat.53032, Active Motif, Shanghai, China) according to the manufacturer’s instructions. Briefly, cells were fixed with 1% formaldehyde, and the reaction was quenched with glycine stop-fix solution. Cells were harvested using cell scraping solution and centrifuged to obtain pellets. The pellets were lysed in lysis buffer and sonicated using a Bioruptor Plus (Diagenode) to shear chromatin into fragments. Chromatin fragments were immunoprecipitated with an *anti*-H3K27me3 antibody (cat. 9733, Cell Signaling Technology). Antibody-bound complexes were captured using magnetic beads and eluted. After cross-link reversal, the immunoprecipitated DNA was purified and used as a template for qPCR.

#### Quantitative RT-PCR

Total RNA was extracted using the RNeasy Mini Kit (Qiagen, 74104). cDNA synthesis and quantitative PCR were performed using the iTaq Universal One-Step RT-qPCR Kit (cat. 1725150, Bio-Rad) on an Applied Biosystems QuantStudio system. Gene expression was normalized to GAPDH expression. The primers used are listed in Table S2.

#### Glucose assay

To measure glucose concentrations, 5 μL of cell lysate, culture medium, or mouse plasma was mixed with 185 μL of Glucose Assay Reagent (cat. S0201S, Beyotime) in a PCR tube to a final volume of 190 μL. The mixture was incubated at 95°C for 8 min and then cooled on ice. After brief vortexing, 180 μL of the reaction mixture was transferred to a 96-well plate. The absorbance was measured at 630 nm, and glucose concentrations were calculated.

#### L-lactate assay

The level of L-lactate was determined using an L-Lactate Assay Kit with WST-8 (cat. S0208SBeyotime). A total of 50 μL of sample was added to the wells of a 96-well plate, followed by the addition of 50 μL of WST-8 chromogenic working solution to each well. After thorough mixing, the plate was protected from light and incubated at 37°C for 30 min. Absorbance was measured at 450 nm. The lactate concentration was calculated based on a standard curve.

#### ATP assay

Cells were seeded and allowed to adhere in opaque-walled multiwell plates. After specific treatments, 100 μL of CellTiter-Glo reagent (cat. G7570, Promega, WI, USA) was added to each well containing 100 μL of culture medium. The plate was mixed on an orbital shaker for 2 min to induce cell lysis and then incubated at room temperature for 10 min to stabilize the luminescent signal. Luminescence was measured using a Synergy microplate reader.

#### cAMP assay

The level of cAMP was assessed using a cAMP Direct Immunoassay Kit (cat. ab138880, Abcam). Cells were grown in a sterile 96-well plate and incubated overnight in a 37°C/5% CO_2_ incubator, followed by treatment as required. After incubation, the culture medium was aspirated. Next, 100 μL of Cell Lysis Buffer was added and incubated at room temperature for 10 min. The lysates were centrifuged at 4°C for 5 min to remove insoluble material. The supernatant was collected, transferred to a new tube, and kept on ice. Subsequently, 5–75 μL of the samples were added to wells of the anti-cAMP coated 96-well plate, and the volume was adjusted to 75 μL with Assay Buffer. The plate was incubated at room temperature for 5–10 min. Then, 25 μL of 1X HRP-cAMP conjugate was added to each well containing standards or samples. The plate was incubated at room temperature for 2 h on a plate shaker. After incubation, the solution was aspirated, and the plate was washed 4 times with 200 μL of 1X Wash Solution per wash. Finally, 100 μL of AbRed Working Solution was added to each well, mixed thoroughly, and incubated at room temperature for 10 min to 2 h protected from light. Fluorescence was measured at excitation/emission of 540/590 nm using a microplate reader in top-read mode. cAMP concentrations were calculated based on the standard curve.

#### NADP^+^/NADPH assay

The level of NADP^+^/NADPH was assessed using an NADP^+^/NADPH Assay Kit with WST-8 (cat. S0179, Beyotime). Cells were collected and lysed by adding an appropriate volume of NADP^+^/NADPH extraction buffer, followed by thorough homogenization. The lysate was centrifuged at 12,000 × g and 4°C for 5–10 min, and the supernatant was collected for analysis. For measurement of total NADP^+^ and NADPH, 50 μL of the supernatant was transferred directly to a 96-well plate. For specific quantification of NADPH, 50 μL of the supernatant was incubated at 60°C for 30 min to decompose NADP^+^. Then, 100 μL of G6PDH working solution was added to each sample and incubated at 37°C for 10 min protected from light. Subsequently, 10 μL of chromogenic solution was added to each well, mixed thoroughly, and incubated at 37°C for 10–20 min protected from light. Absorbance was measured at 450 nm, and the concentrations of NADP^+^ and NADPH were calculated using a standard curve.

#### Pyruvic acid assay

Pyruvate acid concentrations in cell lysates were determined using the Amplex Red Pyruvic Acid Detection Kit (cat. S0299S, Beyotime). The assay was performed following the protocol provided by the manufacturer. In summary, after lysing cells on ice for 5–10 min, the lysates were clarified by centrifugation. A master mix of the Amplex Red working solution was prepared immediately before use. Aliquots of the supernatant or pyruvate standards were dispensed in duplicate into a 96-well plate. The volume in each well was normalized to 20 μL with the provided assay buffer, after which 80 μL of the working solution was added. The reaction mixture was protected from light and incubated at 37°C for 30 min. Fluorescence intensity was measured with a microplate reader at an excitation wavelength of 560 nm and an emission wavelength of 590 nm. Sample pyruvate concentrations were calculated by regression analysis against the standard curve.

#### Cellular glucose-6-phosphate assay

Cellular glucose-6-phosphate (G6P) levels were measured using a G6P Detection Kit with WST-8 (cat. S0185, Beyotime). Briefly, approximately 1 × 10^6^ adherent cells were lysed with 200 μL of ice-cold G6P extraction buffer. Cell lysates and standards (50 μL each) were loaded in duplicate into a 96-well plate. The G6P detection working solution was freshly prepared by mixing chromogenic solution and enzyme mix as directed. Then, 50 μL of detection solution was added to each well, followed by gentle mixing and incubation at 37°C for 10 min in the dark. Absorbance was measured at 450 nm, and incubation time was extended up to 30 min when weak signal was observed. Background correction was performed by subtracting the absorbance of the blank control (0 μM G6P) and, if necessary, sample-specific background controls (samples without enzyme mix). G6P concentrations were calculated based on the standard curve after blank subtraction.

### Quantification and statistical analysis

#### Statistical analysis

Data were analyzed using GraphPad Prism (version 9) and SPSS statistical software. For comparisons between two groups, unpaired two-tailed Student’s *t* test was used. For comparisons among more than two groups, one-way ANOVA or two-way ANOVA with appropriate multiple comparison correction was applied. Diurnal rhythms were analyzed using Cosinor-based circadian curve fitting. Data are presented as mean ± standard deviation (SD), and the n indicates the number of independent experiments conducted using distinct mice, separate cell batches, or distinct tumor tissues. A *p*-value less than 0.05 was considered statistically significant. Mice were randomly assigned to experimental groups before treatment.

## Supplemental information

Document S1. Figures S1–S3

Table S1. Patient characteristics and clinical data

Table S2. Primer sequences used for quantitative RT-PCR
