# Supplementary material for: HHEX-PRKAR2B axis-mediated PKA activation drives glucose metabolism-dependent progression of pancreatic ductal adenocarcinoma
Source: iScience. 2026 Jan 14;29(2):114691. doi: 10.1016/j.isci.2026.114691 (PMC12907050; doi:10.1016/j.isci.2026.114691)
Supplement: Document S1. Figures S1–S3 [file mmc1.pdf]

iScience, Volume *29*

Supplemental information

HHEX-PRKAR2B axis-mediated PKA activation drives glucose metabolism-dependent progression of pancreatic ductal adenocarcinoma

Junxiang Wen, Qiuchen Li, Shuxiang Xu, Wenjun Lu, Fei Wu, Jiatao Lou, and Lin Wang

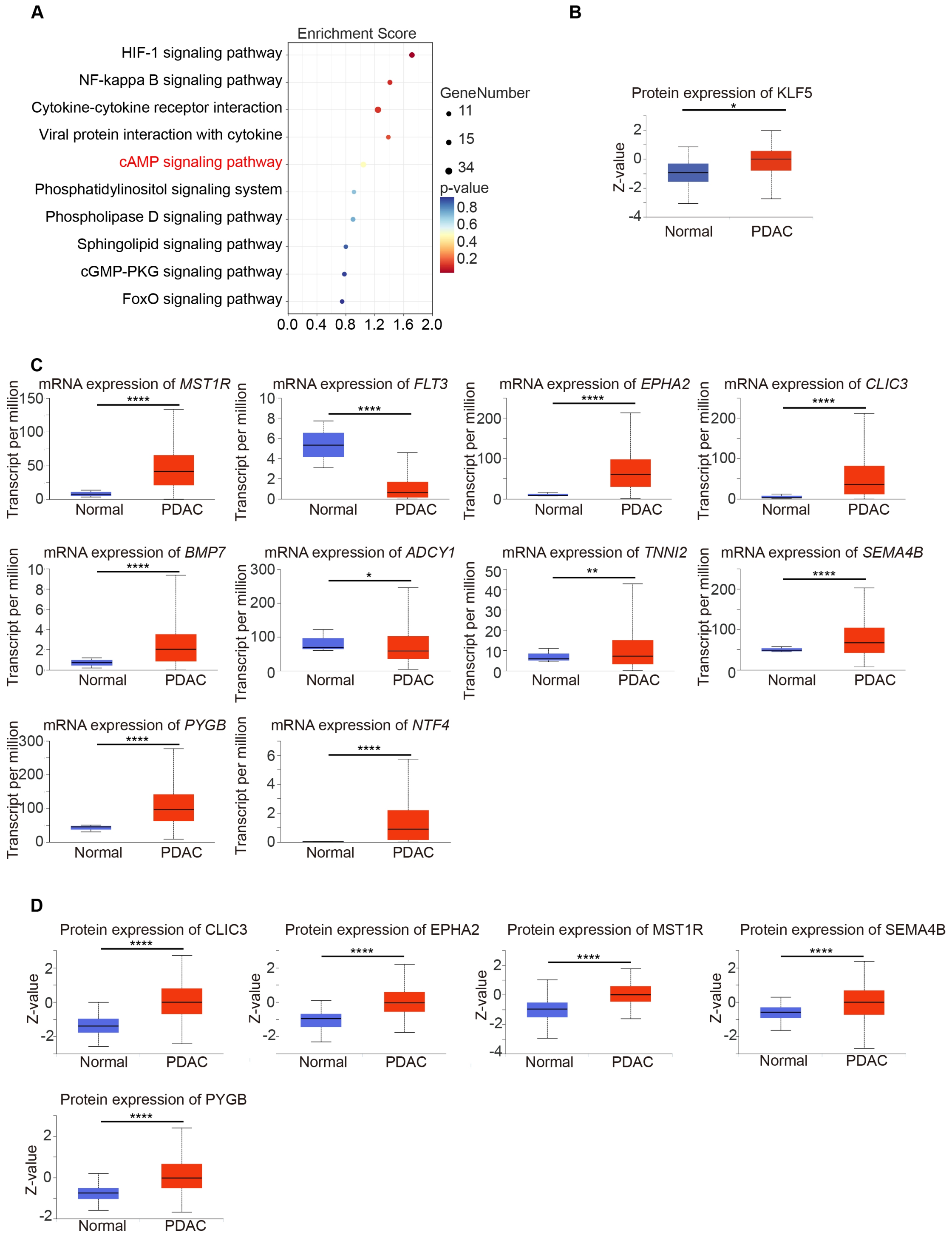
A
Enrichment Score
HIF-1 signaling pathway
NF-kappa B signaling pathway
Cytokine-cytokine receptor interaction
Viral protein interaction with cytokine
cAMP signaling pathway
Phosphatidylinositol signaling system
Phospholipase D signaling pathway
Sphingolipid signaling pathway
cGMP-PKG signaling pathway
FoxO signaling pathway
GeneNumber
11
15
34
p-value
0.8
0.6
0.4
0.2
0.0 0.4 0.8 1.2 1.6 2.0
B
Protein expression of KLF5
Z-value
Normal
PDAC
C
mRNA expression of MST1R
mRNA expression of FLT3
mRNA expression of EPHA2
mRNA expression of CLIC3
mRNA expression of BMP7
mRNA expression of ADCY1
mRNA expression of TNNI2
mRNA expression of SEMA4B
mRNA expression of PYGB
mRNA expression of NTF4
Transcript per million
D
Protein expression of CLIC3
Protein expression of EPHA2
Protein expression of MST1R
Protein expression of SEMA4B
Protein expression of PYGB

Figure S1

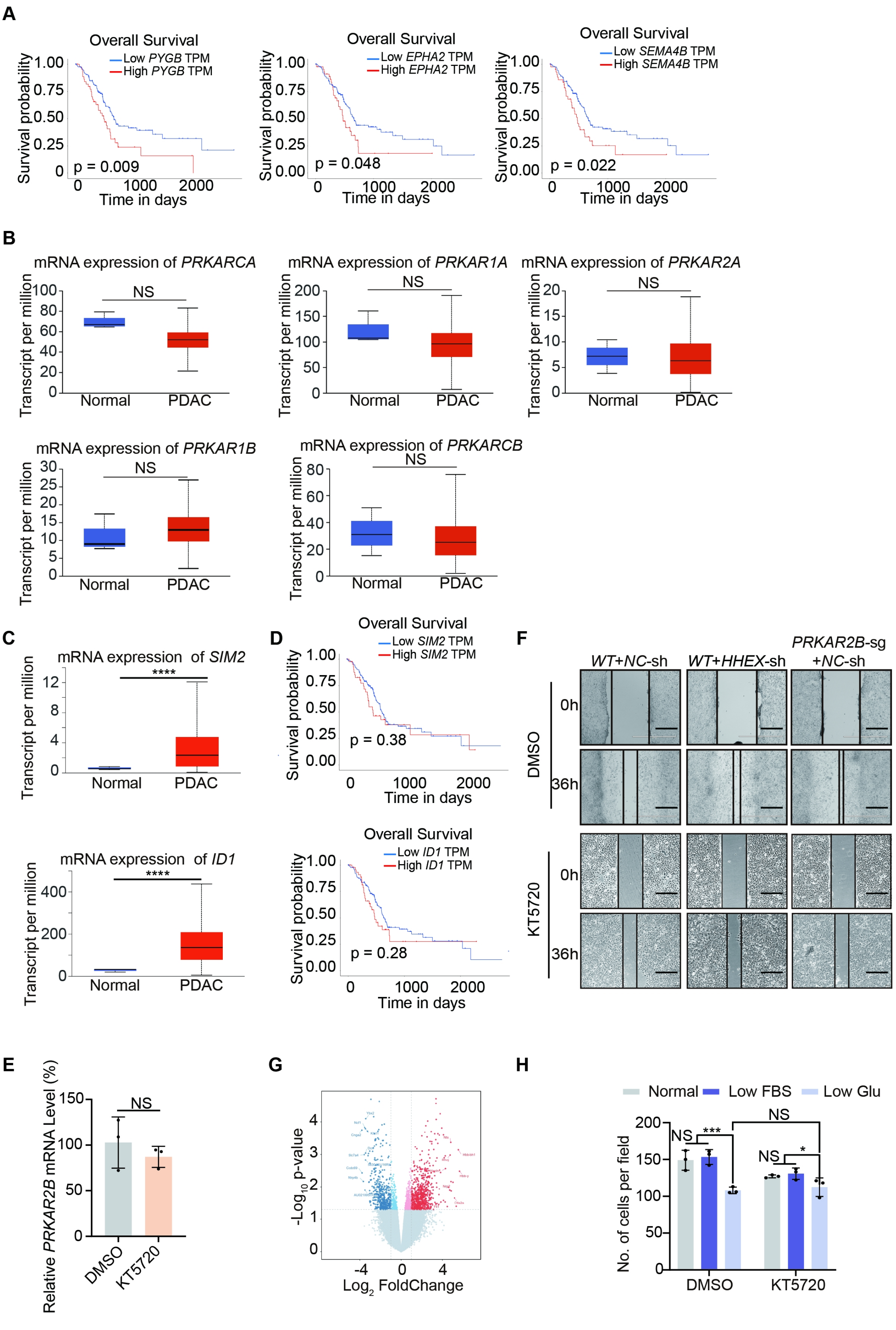
A
Overall Survival
Low PYGB TPM
High PYGB TPM
Survival probability
p = 0.009
Time in days
Overall Survival
Low EPHA2 TPM
High EPHA2 TPM
p = 0.048
Overall Survival
Low SEMA4B TPM
High SEMA4B TPM
p = 0.022
B
mRNA expression of PRKARCA
mRNA expression of PRKAR1A
mRNA expression of PRKAR2A
mRNA expression of PRKAR1B
mRNA expression of PRKARCB
Transcript per million
NS
Normal
PDAC
C
mRNA expression of SIM2
mRNA expression of ID1
****
D
Overall Survival
Low SIM2 TPM
High SIM2 TPM
p = 0.38
Low ID1 TPM
High ID1 TPM
p = 0.28
F
WT+NC-sh
WT+HHEX-sh
PRKAR2B-sg +NC-sh
DMSO
KT5720
0h
36h
E
Relative PRKAR2B mRNA Level (%)
DMSO
KT5720
G
-Log10 p-value
Log2 FoldChange
H
Normal
Low FBS
Low Glu
No. of cells per field
***
*

Figure S2

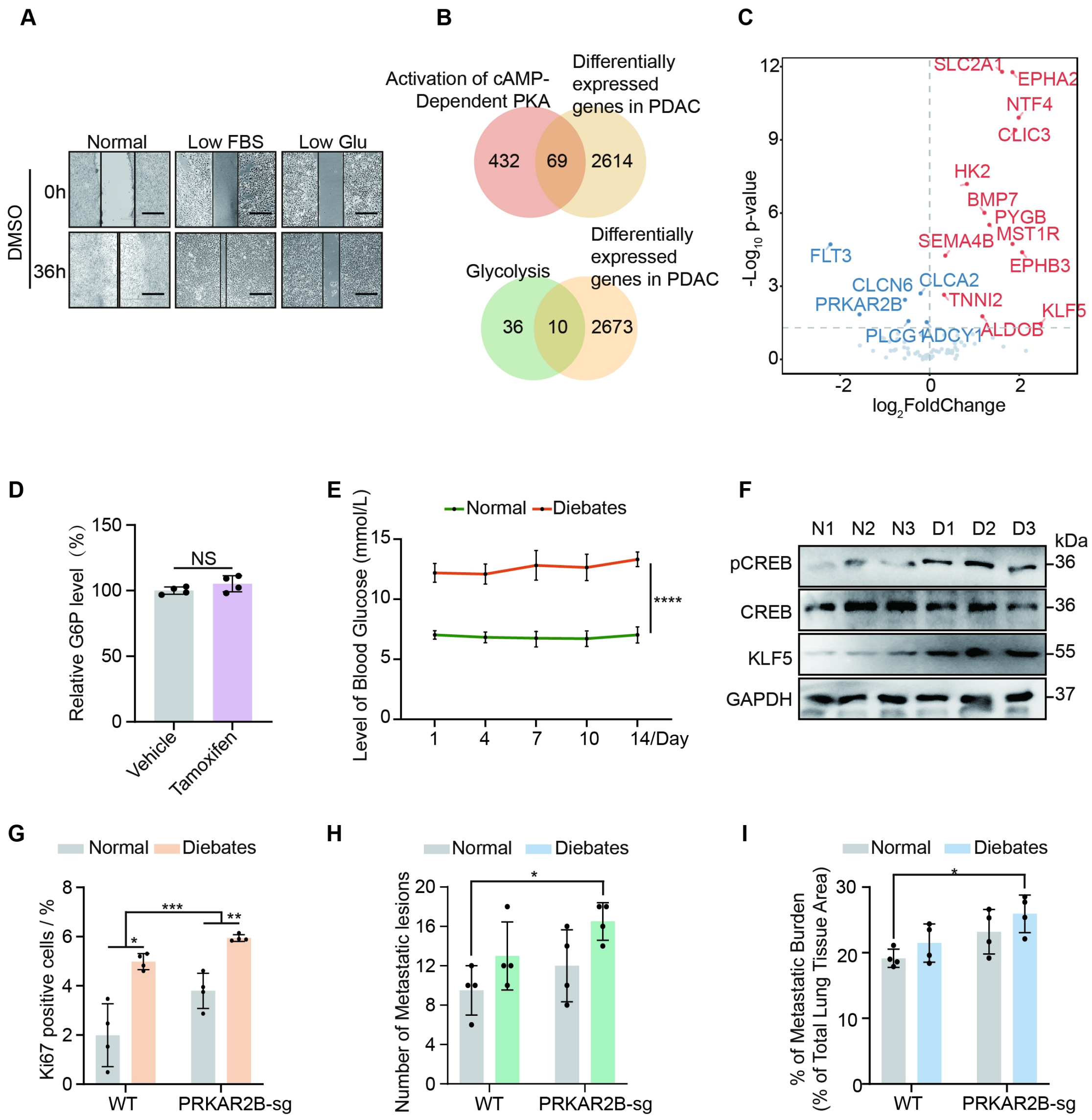

Figure S3

Supplymentary Figure Legend

Figure S1

A. KEGG pathway enrichment analysis of differentially expressed genes from RNA-seq in GEO database (GSE16515). The "cAMP signaling pathway" is highlighted in red.

B. Protein expression levels of KLF5 based on data from 137 PDAC patients and 74 normal control in the CPTAC database. *, $p < 0.05$.

C. mRNA expression levels of indicated genes based on data from 182 PDAC patients and 4 normal control in the TCGA database. *, $p < 0.05$; **, $p < 0.01$; ****, $p < 0.0001$.

D. Protein expression levels of indicated genes based on data from 137 PDAC patients and 74 normal control in the CPTAC database. ****, $p < 0.0001$.

Figure S2

A. Kaplan–Meier survival analysis of indicated genes expression using data from 182 PDAC

patients in the TCGA database. *, $p < 0.05$; **, $p < 0.01$.

B-C. mRNA expression levels of indicated genes based on data from 182 PDAC patients and 4 normal control in the TCGA database. ****, $p < 0.0001$; NS, no significance.

D. Kaplan – Meier survival analysis of indicated genes expression using data from 182 PDAC patients in the TCGA database. *, $p < 0.05$; **, $p < 0.01$.

E. mRNA expression levels of *PRKAR2B* in AsPC-1 cells with or without KT5720 treatment (n = 3 independent repeats). Data were expressed as mean ± SD. NS, no significance.

F. Wound healing assays performed in AsPC-1 cells following *HHEX* knockdown or *PRKAR2B* knockout with or without KT5720 treatment (5 μM, 48 hours). Scale bars, 300 μm.

G. Volcano plot of RNA-seq data from ES cells with or without doxycycline induction of constitutively active PKA (GSE134618). Red dots indicate upregulated mRNAs ($p < 0.05$, $\log_2$FoldChange > 1 or < -1); blue dots indicate downregulated mRNAs.

H. The bar chart shows the number of invaded cells per field in figure 4J. Data were expressed as mean ± SD (n = 3 independent repeats) . Scale bars, 80 μm. *, $p < 0.05$, ***, $p < 0.001$; NS, no significance.

Figure S3

A. Wound healing assays performed in AsPC-1 cells treated with low FBS (2%) or low glucose (1000 mg/L). Scale bars, 300 μm.

B. Venn diagram depicting the intersection among differentially expressed genes in PDAC (from dataset GSE226307) and gene sets associated with 'activation of cAMP-dependent PKA' and 'glycolysis' (sourced from the PathCard database).

C. Volcano plot of the 79 overlapping genes from Supplementary Figure S3B, analyzed from a comparison of 182 PDAC tumors versus 4 normal samples in the TCGA database. Red dots indicate upregulated mRNAs ($p < 0.05$); blue dots indicate downregulated mRNAs.

D. Relative glucose-6-phosphate (G6P) levels in pancreatic tissue compared between tamoxifen-injected and vehicle-injected mice with pancreas-specific KRAS expression (n = 4 independent samples). Data were expressed as mean ± SD. NS, no significance.

E. Blood glucose levels in wild-type and diabetic mice (n = 4 independent samples). Data were expressed as mean ± SD. ****, $p < 0.0001$.

F. Representative immunoblot of CREB, pCREB, and KLF5 in wild-type (N) and diabetic mice (D) (n = 4 independent samples; see Figure 6J for all replicates).

G. The bar chart shows the persentage of Ki67 positive cells in figure 6O. *, $p < 0.05$; **, $p < 0.01$; ***, $p < 0.001$.

H-I. Quantification of metastatic lesion number (H) and total metastatic burden (I) at the experimental endpoint across the indicated treatment groups (n = 4 independent samples). Data were expressed as mean ± SD. *, $p < 0.05$.
